# Dementia risk by metabolic health and obesity in two prospective cohorts

**DOI:** 10.1186/s12916-026-05002-8

**Published:** 2026-06-19

**Authors:** Martin Nakash, Elsa Ojalehto Lindfors, Yiqiang Zhan, Anna K. Dahl Aslan, Chandra A. Reynolds, Peggy Ler, Ida K. Karlsson

**Affiliations:** 1 Department of Medical Epidemiology and Biostatistics, Karolinska Institutet, Stockholm, Sweden; 2 School of Public Health (Shenzhen), Sun Yat-sen University, Shenzhen, China; 3 School of Health Sciences, University of Skövde, Skövde, Sweden; 4 Institute for Behavioral Genetics, Department of Psychology and Neuroscience, University of Colorado Boulder, Boulder, CO USA; 5 Nutrition and Metabolism Branch, International Agency for Research on Cancer, Lyon, France

**Keywords:** Obesity, Dementia, Metabolic health, Obesity and metabolic health phenotypes

## Abstract

**Background:**

Midlife obesity is a well-established risk factor for dementia, whereas late-life obesity has been associated with no increased risk, or even a reduced risk in some studies. However, the joint associations of obesity (body mass index ≥ 30 kg/m^2^) and metabolic health phenotypes (defined by the presence of hyperglycemia, hypertension and dyslipidemia) with dementia risk are less explored, particularly with regard to age- and sex-related differences. Therefore, we investigated how obesity and metabolic health phenotypes jointly associate with dementia risk and whether this risk differs between midlife (≤ 65 years) and late-life (> 65 years), and sex.

**Methods:**

We analysed data from 11,482 participants, aged 51 to 100 years, from the US Health and Retirement Study (HRS), and 13,068 participants, aged 45 to 90 years, from the Swedish Twin Registry (STR). Cox regression models were used to estimate dementia risk in relation to metabolically healthy obesity (MHO), metabolically unhealthy obesity (MUO), and metabolically unhealthy no obesity (MUNO), relative to metabolically healthy no obesity (reference). Models were adjusted for age, sex, smoking status, and education level. Analyses were stratified by midlife and late-life and conducted in the entire sample and separately by sex.

**Results:**

Metabolically unhealthy status in midlife and late-life indicated increased dementia risk regardless of obesity status, reaching statistical significance for midlife MUNO in females in HRS (Hazard ratio (HR): 1.62, 95% confidence intervals (CI): 1.05–2.49) and in late-life MUNO for the full sample in STR (HR: 1.13, CI: 1.02–1.25) and males in the STR (HR: 1.22, CI: 1.04–1.42). The associations between MUO and dementia risk were not statistically significant, but trends suggested midlife MUO was associated with higher dementia risk. The associations between mid and late-life MHO with dementia risk were also not statistically significant, although the associations showed trends towards lower dementia risk.

**Conclusions:**

Being metabolically unhealthy, especially in midlife, may be associated with increased dementia risk, regardless of obesity status. Mid- and late-life MHO showed no increased risk and suggested potential inverse associations. These findings underscore the importance of evaluating dementia risk in the context of obesity, metabolic health, age and sex simultaneously.

**Supplementary Information:**

The online version contains supplementary material available at 10.1186/s12916-026-05002-8.

## Background

Obesity in midlife is an established risk factor for dementia [1]. However, the association between obesity in late-life and dementia is inconclusive: some previous studies found no association, others reported that late-life obesity had an increased dementia risk, while some found a reduced risk [2]. The latter suggests an “obesity paradox”, whereby obesity in midlife is associated with an increased risk of dementia, but a high body mass index (BMI) in late-life is associated with no risk or even a reduced risk [1, 2].

The association between obesity and dementia risk is nuanced and may differ across obesity phenotypes based on metabolic profiles. While individuals with obesity often present with metabolic dysfunction, some do not, leading to the classification of the metabolically healthy obesity (MHO) phenotype [3]. Individuals with MHO have higher risks of adverse health outcomes compared to metabolically healthy normal weight, but better health outcomes compared to metabolically unhealthy obesity (MUO) [4]. A recent systematic review and meta-analysis on MHO and dementia risk reported a lower risk of dementia in MHO compared to individuals without obesity who were metabolically healthy, whereas MUO showed no clear association [5]. Importantly, individuals without obesity (BMI < 30 kg/m^2^) who were metabolically unhealthy had the highest risk of dementia [5]. However, the three studies included in the meta-analysis all used data from late-life samples, and the associations may in part reflect the obesity paradox, with inverse associations between late-life obesity and dementia risk [2, 5–8]. The relatively few studies based on midlife measures of obesity and metabolic health indicate a higher risk of dementia for individuals who are metabolically unhealthy, regardless of obesity status, and potentially, also individuals with MHO [8–10]. One prospective cohort study examined age-specific associations and showed that individuals < 60 years old had a higher risk of dementia if they had obesity, regardless of metabolic health status, compared to being metabolically healthy without obesity [9]. In addition, being metabolically unhealthy was associated with higher dementia risk also in the no obesity category. At higher ages, all associations were attenuated, but without indications of a lower dementia risk in those with MHO, even at age 70 years or above [9]. Taken together, differences in associations may reflect the obesity paradox and highlight the importance of accounting for age at the measurement of obesity and metabolic health status when investigating the association between MHO and dementia risk.

The current study aimed to improve our understanding of how both obesity and metabolic health are associated with dementia risk, and whether these associations differ depending on whether measurements were taken in midlife or late-life. As few previous studies have investigated sex differences in the association between MHO and dementia, we additionally aimed to test the associations separately in males and females. To do so, we used data from the Health and Retirement Study (HRS) in the United States, and the Swedish Twin Registry (STR), with objectively measured obesity and metabolic health markers and prospective dementia information. We hypothesized that in midlife, a metabolically unhealthy status regardless of obesity status, as well as MHO, would be associated with a higher risk of dementia, reflecting the adverse health effects of midlife obesity and metabolically unhealthy status. In late-life, we hypothesized that being metabolically unhealthy would remain associated with higher risk of dementia regardless of obesity status, while MHO would be associated with a lower risk, reflecting the obesity paradox while also considering adverse effects of metabolic dysfunction.

## Methods

This prospective cohort study is based on two population-based longitudinal cohorts: HRS [11] and the STR [12]. The HRS is a cohort of individuals aged 50 and above from the United States, along with their spouses, with biennial follow-up since 1992 [11]. HRS conducted extensive interviews with study participants, covering topics such as demographics, socioeconomic circumstances, work life, environment, and lifestyle [11]. Until 2004, interviews were conducted mainly by telephone. Since 2006, half of the participants have been interviewed by phone and the other half by face-to-face interviews, with alternating waves such that participants undergo face-to-face interviews every 4 years [11]. The face-to-face interviews included the same information as the telephone interviews, as well as the collection of additional blood samples and physical measurements [11]. The HRS consists of 43,695 individuals, of whom 22,377 participated in at least one face-to-face interview between 2006 and 2016 and were included in the current study.

**Fig. 1:**
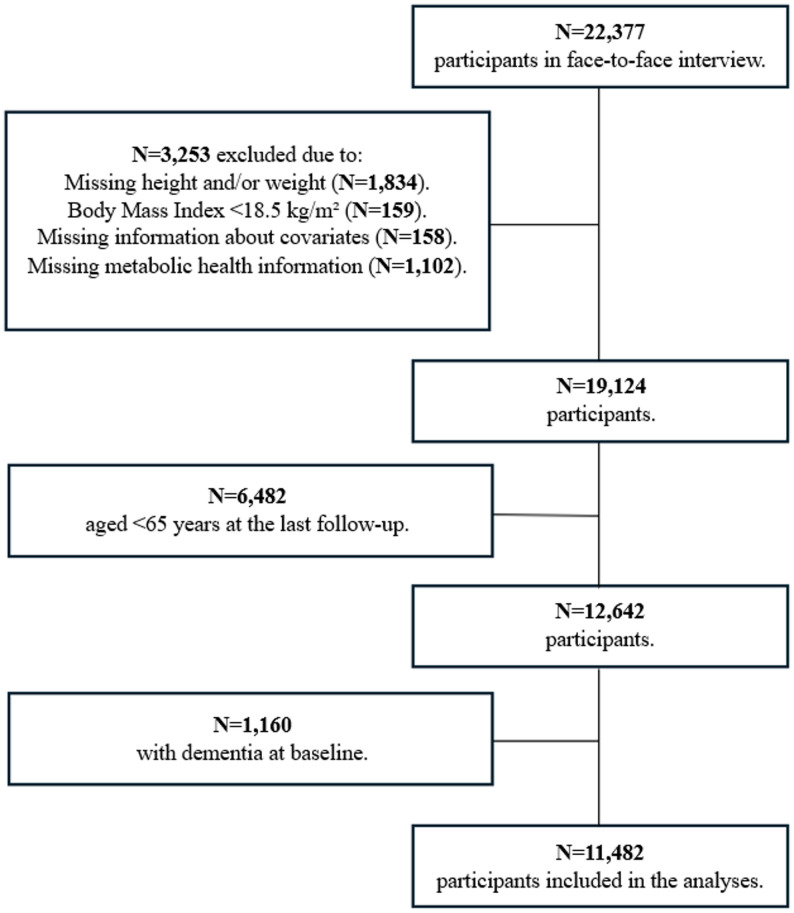
Flow chart of the Health and Retirement Study sample. The flow chart shows the exclusion criteria and the number of individuals from the Health and Retirement Study included in the current study

**Fig. 2:**
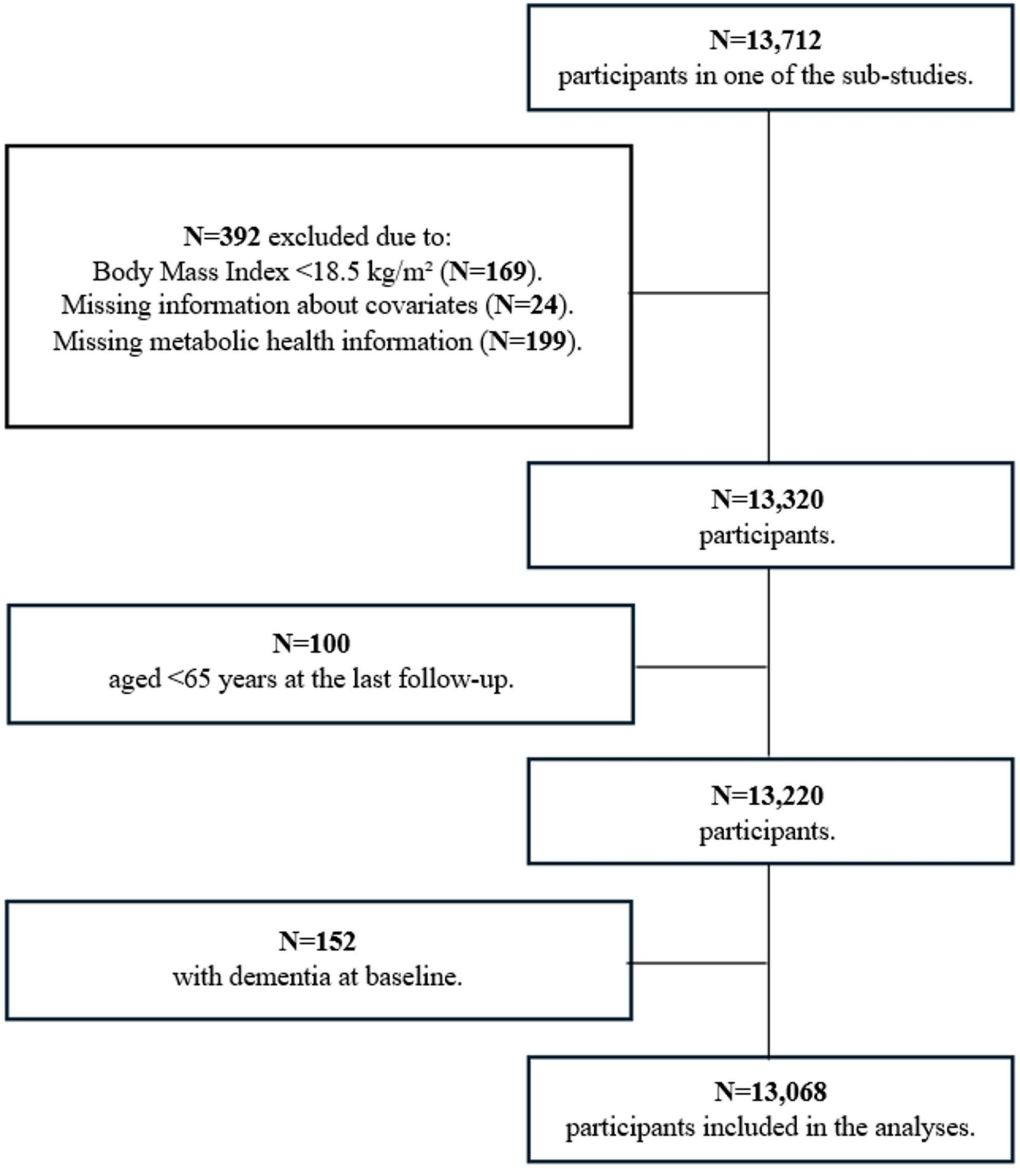
Flow chart of the Swedish Twin Registry sample. The flow chart shows the exclusion criteria and the number of individuals from the Swedish Twin Registry included in the current study

The STR was established in the 1960s and contains information on approximately 87,000 twin pairs born in Sweden between 1886 and 2008 [12]. For the current study, we included data from four sub-studies within the STR: The Swedish Adoption/Twin Study of Aging (SATSA), Aging in Women and Men (GENDER), Origins of Variance in the Oldest Old: Octogenarian Twins (OCTO-Twin), and TwinGene. SATSA was a longitudinal study of 859 individuals from same-sex twin pairs, with up to 10 in-person assessments over a period of approximately 30 years (1986–2014) [13]. GENDER was initiated in 1995 and involved 248 pairs of opposite-sex twins who participated in up to three in-person assessments on a 4-year rolling schedule [14]. OCTO-twin included twins aged 80 and older, with 351 same-sex twin pairs participating in up to five assessments at 2-year intervals, starting in the year 1991 [15]. TwinGene was a cross-sectional study in which 12,630 twins born before 1959 completed a questionnaire and participated in a health examination between 2004 and 2008 [12]. The sample stems from the Screening Across the Lifespan Twin Study (SALT), an extensive telephone interview aimed at all Swedish twins conducted 1998–2002 from where survey data were available [12]. In total, 13,712 individuals participated in at least one of the four sub-studies and were included in the current study. Figures 1 and 2 present flow charts of individuals from the HRS and STR included in the analyses.

### Outcome: dementia

Individuals with dementia at baseline were excluded. In the HRS, dementia was classified according to the Langa-Weir Classification of Cognitive Function, validated for observational studies [16]. In the interviews, all HRS respondents were asked a series of questions, and scored on a 27-point (range 0–27) scale used to determine total cognitive functioning [16]. This scale measures: (1) memory by immediate and delayed 10-noun free recall test (0 to 20 points); (2) working memory by a serial sevens subtraction test (0 to 5 points); and (3) speed of mental processing by a counting backwards test (0 to 2 points) [16]. The cut-points of the 27-point scale were: Normal cognition, 12–27 points; Cognitive Impairment but no Dementia (CIND), 7–11 points; and Dementia, 0–6 points [16]. Individuals categorized as CIND but never as dementia were included in the analyses, but were censored when categorized as CIND.

In the STR, dementia diagnoses were identified through linkages to national healthcare registers and assessments part of the longitudinal sub-studies, as described in detail elsewhere [17]. Briefly, using the unique personal identification number assigned to all Swedish residents, diagnoses were obtained from the National Patient Register (NPR) and the Cause of Death Register (CDR) [18]. Prescribed dementia medication from the Prescribed Drug Register was used as a proxy for the diagnoses [17]. Data from all three registers were available with linkage through 2023. Diagnostic and therapeutic codes used to define dementia are provided in Additional file 1: Tables S1 and S2 [17, 18]. In addition, dementia was assessed during in-person testing in the SATSA, GENDER and OCTO-Twin studies [17]. This included a comprehensive cognitive test battery, with suspicion of dementia followed by review of medical records and final diagnosis set through multidisciplinary consensus conferences [17].

### Exposure: obesity and metabolic health

Obesity was defined based on BMI, calculated as a person’s weight in kilograms divided by the square of height in meters. A BMI of ≥ 30 kg/m2 was considered obesity, and a BMI of < 30 kg/m2 was considered no obesity. While no consensus exists, MHO is typically defined based on the definition of metabolic syndrome, which is to not have more than one of four criteria: hyperglycemia, hypertension, dyslipidemia based on high-density lipoprotein cholesterol (HDL-C), and dyslipidemia based on triglycerides (TG) [3]. Since TG was not measured in the HRS, a metabolically unhealthy status was defined as having at least two of the other three metabolic health variables: hyperglycemia, hypertension, or dyslipidemia based on HDL-C levels in HRS. In STR, a metabolically unhealthy status was defined as having at least two of the four aforementioned metabolic health variables. For comparability with the HRS, sensitivity analyses defined metabolic health based on hyperglycemia, hypertension, and dyslipidemia based on HDL-C in the STR (excluding TG). In addition, a stricter criterion was tested in sensitivity analyses, defining unhealthy status as only having one of the criteria in both cohorts. The diagnostic criteria for the metabolic health variables are presented in Additional file 1: Table S3.

Obesity and metabolic health phenotypes were categorized as follows: (1) metabolically healthy no obesity (MHNO), reference category, with a BMI of < 30 kg/m2 and being metabolically healthy; (2) metabolically unhealthy no obesity (MUNO), with a BMI of < 30 kg/m2 and being metabolically unhealthy; (3) MHO, with a BMI of ≥ 30 kg/m2 and being metabolically healthy; and (4) MUO, with a BMI of ≥ 30 kg/m2 and being metabolically unhealthy.

### Covariates

For the HRS data, covariates were retrieved from the HRS core data and the RAND HRS Longitudinal File 2022 (V1), an easy-to-use dataset based on the HRS core data. In the STR, covariates were retrieved from the respective study, alternatively from the SALT interview. We included the following covariates: age in years at measurement, sex (male or female), smoking status (currently smoking or not smoking), and education level. In the HRS, education level was categorized into five categories: less than a high school education, general educational development (GED), high school graduate, some college, and college and above. In the STR, education level was categorized into basic education (≤ 7 years) or higher education (> 7 years). In the HRS, ethnicity was also considered, categorized as White/Caucasian, Black/African American or other. In the STR, sub-study was considered (SATSA, OCTO-twin, GENDER or TwinGene).

Additionally, sensitivity analyses further adjusted for physical activity, alcohol consumption, depressive symptoms, and Apolipoprotein E (*APOE)* ε4 genotype. In the HRS, physical activity was assessed as vigorous (such as running or jogging, swimming, cycling, aerobics or gym workout, tennis, or digging with a spade or shovel) and moderate-intensity (gardening, cleaning the car, walking at a moderate pace, dancing, floor or stretching exercises) physical activity, both categorized into five levels: “never”, “1–3 times per month”, “once per week”, “more than once per week”, and “every day”. A summary score of the two was included in the analyses. In the STR, a three-level variable was created based on questions in SATSA, GENDER, and SALT (as described in Additional File 1: Table S4). In the HRS, alcohol consumption was assessed as drinks per week, based on questions on the average number of days per week the respondent had any alcohol to drink, and about how many drinks they had on those days. In the STR, we used a harmonized variable available through the Interplay of Genes and Environment across Multiple Studies (IGEMS) consortium, measuring grams of alcohol per week [19, 20]. Depressive symptoms were assessed using the Center for Epidemiologic Studies Depression (CES-D) scale in all studies. In the HRS, a revised summary score was constructed to reflect respondents’ feelings during the week prior to the interview, comprising a summary score of binary responses to six negative indicators and the inverse of two positive indicators [21]. In the STR, the CES-D was administered in SATSA, OCTO-Twin, and GENDER and the shortened 11-item CES-D scale in SALT, and we used a depressive symptoms score harmonized by the IGEMS consortium [20, 22, 23]. *APOE* ε4 was directly genotyped in the HRS (TaqMan allelic discrimination assays) and in SATSA and GENDER (Illumina GoldenGate assays) [24, 25]. For TwinGene participants, genome-wide genotyping data were available from the Human OmniExpress array, and *APOE *ε4 genotype inferred from 1000 genomes imputed data, using established protocols [12, 26]. Individuals were categorized into carriers and non-carriers of the *APOE* ɛ4 allele.

### Statistical analyses

Descriptive statistics were performed on the midlife and late-life sample of both cohorts, and presented for the total analytical samples and by dementia status. Mean and standard deviation were calculated for continuous variables and frequency and percentage for categorical variables.

In primary analyses, Cox proportional hazard regression was applied to model the risk of dementia in relation to MHO, MUO, and MUNO, compared to the reference category MHNO [27]. Age was used as the underlying timescale, and individuals were followed from age at first available measure of obesity and metabolic health to either age at dementia (first wave classified as dementia in the HRS, age at onset or diagnosis in the STR), death, or end of follow-up (last wave of participation for the HRS (year 2020), end of register follow-up for the STR (year 2023)), whichever occurred first. In the HRS, controls were further censored at the first wave categorized as CIND (those later categorized as having dementia were not censored at CIND, as CIND is considered part of the progression to dementia). To account for non-independence in the data, we used robust standard errors to adjust for clustering at the twin-pair level in the STR and at the household level in the HRS. All models were adjusted for sex, education level, and smoking status. To allow for differences in the baseline hazard, stratified Cox models were applied, stratifying by ethnicity in the HRS and by sub-study in the STR.

Models were applied separately for BMI and metabolic health measures taken in midlife, ages ≤ 65 years, or late-life, ages > 65 years, to the total analytical sample, and separately to the male and female analytical sample. The assumption of proportional hazards was examined through Schoenfeld’s residuals [28], which did not indicate any violations, with the exception of the late-life sample in the HRS where the education variable indicated non-proportional hazards and was included in the strata statement.

In secondary analyses, we repeated the analyses modelling obesity and metabolic health separately, to better understand their respective associations with dementia. We modeled (1) only obesity, (2) only metabolic health, (3) obesity and metabolic health jointly (mutually adjusted for), and (4) an interaction between obesity and metabolic health.

### Sensitivity analyses

To evaluate the robustness of the findings, several sensitivity analyses were performed. First, individuals aged 63–67 years were excluded to assess potential bias from misclassification around age cutoffs, leading to obscured results reflecting the obesity paradox. Second, since there is no agreement what constitutes metabolic health, the definition of metabolically unhealthy status was modified so that having one (instead of two) of the metabolic health variables was enough to be considered metabolically unhealthy [4]. Third, as metabolic health criteria differed between the STR and the HRS, we defined metabolic health based on only three criteria also in the STR, excluding TG (hyperglycemia, hypertension, and dyslipidemia based on HDL-C). Fourth, to examine overweight as well as obesity, the exposure variable was categorized into six metabolic health and body weight groups: metabolically healthy normal weight and metabolically unhealthy normal weight, with a BMI between ≥ 18.5 kg/m^2^ and < 25 kg/m^2^; metabolically healthy overweight and metabolically unhealthy overweight, with a BMI between ≥ 25 kg/m^2^ and < 30 kg/m^2^; and metabolically healthy obesity and metabolically unhealthy obesity, with a BMI of ≥ 30 kg/m^2^. Fifth, we further adjusted the models for lifestyle factors, specifically physical activity and alcohol consumption, then additionally for depressive symptoms, and finally for *APOE *ε4 genotype. Sixth, we applied Fine-Gray subdistribution hazards regression to examine the associations, with dementia as the main outcome of interest and death as the competing event in the midlife and late-life group. Age was used as the underlying as the underlying timescale and adjusted as in the primary Cox analyses, with the exception that ethnicity (in the HRS) and study (in the STR) were included as covariates and not as stratification variables. The statistical analyses in this study were conducted in R 4.3.2 through the software RStudio 2023.09.0 [29] using the following key packages: survival [30], multiwayvcov [31], and sandwich [32]. This study was approved by the Swedish Ethical Review Authority in Stockholm (2024-03706-01).

## Results

### Descriptive characteristics of the study population

Descriptive characteristics of the midlife and late-life study populations, both the total analytical sample and stratified by dementia status, are presented in Table 1 for the HRS, and in Table 2 for the STR.

**Table 1:** Descriptive characteristics of the Health and Retirement Study sample

	Midlife study population (≤ 65)			Late-life study population (> 65)		
	Total analytical sample	Dementia	No dementia	Total analytical sample	Dementia	No dementia
Incidence rate, cases per 1000 person-years	8.8			33.0		
**N (%)**	4,233	362 (8.6)	3,871 (91.4)	8,841	2,078 (23.5)	6,763 (76.5)
**Female sex**, N (%)	2,518 (59.5)	201 (55.5)	2,317 (59.9)	5,082 (57.5)	1,231 (59.2)	3,851 (56.9)
**Age in years at baseline**, mean, (SD)	59.7 (3.3)	60.8 (3.0)	59.6 (3.3)	73.2 (6.6)	76.5 (7.2)	72.3 (6.1)
**Age in years at last follow-up** (mean, (SD))	69.4 (3.5)	69.5 (3.5)	69.4 (3.5)	80.4 (7.0)	83.5 (7.3)	79.4 (6.6)
**Ethnicity** N (%)						
White/Caucasian	3,030 (71.6)	175 (48.3)	2,855 (73.8)	7,363 (83.3)	1,509 (72.6)	5,854 (86.6)
Black/African American	802 (18.9)	125 (34.5)	677 (17.5)	1,151 (13.0)	471 (22.7)	680 (10.1)
Other	401 (9.5)	62 (17.1)	339 (8.8)	327 (3.7)	98 (4.7)	229 (3.4)
**Current smoker** N (%)	710 (16.8)	104 (28.7)	606 (15.7)	862 (9.8)	195 (9.4)	667 (9.9)
**Education level** N (%)						
Less than high school	573 (13.5)	170 (47.0)	403 (10.4)	1,735 (19.6)	839 (40.4)	896 (13.2)
GED	203 (4.8)	20 (5.5)	183 (4.7)	403 (4.6)	100 (4.8)	303 (4.5)
High school graduate	1,067 (25.2)	86 (23.8)	981 (25.3)	2,834 (32.1)	584 (28.1)	2,250 (33.3)
Some college	1,190 (28.1)	63 (17.4)	1,127 (29.1)	1,915 (21.7)	321 (15.4)	1,594 (23.6)
College and above	1,200 (28.3)	23 (6.3)	1,177 (30.4)	1,954 (22.1)	234 (11.3)	1,720 (25.4)
**BMI and metabolic health measures**						
BMI in kg/m^2^ (mean, (SD))	30.5 (6.0)	30.7 (6.0)	30.5 (6.0)	29.5 (5.6)	29.0 (5.6)	29.7 (5.6)
Systolic BP (mean, (SD))	127.7 (18.8)	131.7 (20.7)	127.3 (18.6)	132.7 (20.4)	135.6 (21.9)	131.8 (19.8)
Diastolic BP (mean, (SD))	81.3 (11.1)	82.7 (12.5)	81.1 (11.0)	78.6 (11.4)	78.3 (12.0)	78.7 (11.2)
HbA1c in % (mean, (SD))	5.9 (1.1)	6.2 (1.4)	5.8 (1.1)	5.9 (0.9)	6.0 (1.0)	5.8 (0.9)
HDL-C level in mg/dL (mean, (SD))	55.2 (16.5)	52.0 (15.4)	55.5 (16.5)	54.4 (16.1)	53.6 (15.9)	54.7 (16.1)
**Metabolic health and weight phenotypes** N (%)						
MHNO	1,480 (35.0)	86 (23.8)	1,394 (36.0)	2,702 (30.6)	586 (28.2)	2,116 (31.3)
MHO	839 (19.8)	57 (15.7)	782 (20.2)	1,134 (12.8)	213 (10.3)	921 (13.6)
MUNO	726 (17.2)	90 (24.9)	636 (16.4)	2,463 (27.9)	700 (33.7)	1,763 (26.1)
MUO	1,188 (28.0)	129 (35.6)	1,059 (27.4)	2,542 (28.8)	579 (27.9)	1,963 (29.0)

Descriptive statistics for the Health and Retirement Study sample with measures taken in midlife (ages ≤ 65 years) or late-life (ages > 65 years), for the total analytical sample and by dementia status. Numbers are presented as mean and standard deviation of continuous variables and numbers and percentages of categorical variables

Abbreviations: BMI – Body mass index, BP – Blood pressure, GED – General Educational Development, HbA1c – Hemoglobin A1c, HDL-C – High-Density Lipoprotein Cholesterol, MHNO – Metabolically healthy no obesity, MHO – Metabolically healthy obesity, MUNO – Metabolically unhealthy no obesity, MUO – Metabolically unhealthy obesity, N – Number of individuals, SD – Standard deviation

**Table 2:** Descriptive characteristics of the Swedish Twin Registry sample at baseline

	Midlife study population (≤ 65)			Late-life study population (> 65)				
	Total analytical sample	Dementia	No dementia		Total analytical sample	Dementia	No dementia	
Incidence rate, cases per 1000 person-years	3.4				18.4			
**N (%)**	5,856	327 (5.6)	5,529 (94.4)		7,212	1,669 (23.1)	5,543 (76.9)	
**Female sex** N (%)	3,266 (55.8)	181 (55.4)	3,085 (55.8)		3,863 (53.6)	959 (57.5)	2,904 (52.4)	
**Age in years at baseline** (mean, (SD))	59.1 (3.9)	60.5 (3.3)	59.0 (3.9)		72.4 (6.1)	73.9 (5.9)	71.9 (6.0)	
**Age in years at last follow-up** (mean, (SD))	75. 7 (4.8)	76.6 (5.9)	75.7 (4.7)		85.0 (6.0)	83.9 (6.0)	85.3 (5.9)	
**Current smoker** N (%)	3,725 (63.6)	201 (61.5)	3,524 (63.7)		3,730 (51.7)	789 (47.3)	2,941 (53.1)	
**Education level** N (%)								
≤ 7 years of education	1,392 (23.8)	94 (28.7)	1,298 (23.5)		3,021 (41.9)	743 (44.5)	2,278 (41.1)	
> 7 years of education	4,464 (76.2)	233 (71.3)	4,231 (76.5)		4,191 (58.1)	926 (55.5)	3,265 (58.9)	
**BMI and metabolic health measures**								
BMI in kg/m^2^ (mean, (SD))	26.0 (3.9)	25.9 (3.5)	26.0 (4.0)		26.1 (3.8)	25.9 (3.6)	26.1 (3.9)	
Systolic BP (mean, (SD))	135.0 (18.3)	139.9 (19.8)	134.7 (18.2)		146.7 (21.2)	147.4 (21.4)	146.5 (21.1)	
Diastolic BP (mean, (SD))	82.8 (10.5)	84.4 (10.5)	82.7 (10.5)		81.7 (10.7)	81.3 (11.0)	81.9 (10.6)	
HbA1c in % (mean, (SD))	4.7 (0.6)	4.8 (0.9)	4.7 (0.6)		4.9 (0.7)	4.9 (0.7)	4.9 (0.7)	
Glucose level in mmol/L (mean, (SD))	4.9 (1.3)	4.8 (1.5)	4.9 (1.3)		5.5 (2.2)	5.5 (1.8)	5.6 (2.4)	
HDL-C level in mmol/L (mean, (SD))	1.4 (0.4)	1.4 (0.4)	1.4 (0.4)		1.4 (0.4)	1.4 (0.4)	1.4 (0.4)	
Triglycerides in mmol/L (mean, (SD))	1.3 (0.8)	1.4 (0.8)	1.3 (0.8)		1.5 (0.8)	1.5 (0.9)	1.5 (0.8)	
**Metabolic health and weight phenotypes** N (%)								
MHNO	3,404 (58.1)	192 (58.7)	3,212 (58.1)		3,483 (48.3)	806 (48.3)	2,677 (48.3)	
MHO	298 (5.1)	11 (3.4)	287 (5.2)		318 (4.4)	59 (3.5)	259 (4.7)	
MUNO	1,622 (27.7)	94 (28.7)	1,528 (27.6)		2,745 (38.1)	680 (40.7)	2,065 (37.3)	
MUO	532 (9.1)	30 (9.2)	502 (9.1)		666 (9.2)	124 (7.4)	542 (9.8)	

Descriptive statistics for the Swedish Twin Registry sample with measures taken in midlife (ages ≤ 65 years) or late-life (ages > 65 years), for the total analytical sample and by dementia status. Numbers are presented as mean and standard deviation of continuous variables and numbers and percentages of categorical variables

Abbreviations: BMI – Body mass index, BP – Blood pressure, HbA1c – Hemoglobin A1c, HDL-C – High-Density Lipoprotein Cholesterol, MHNO – Metabolically healthy no obesity, MHO – Metabolically healthy obesity, MUNO – Metabolically unhealthy no obesity, MUO – Metabolically unhealthy obesity, N – Number of individuals, SD – Standard deviation

In the HRS sample, 4,233 individuals had BMI and metabolic health measures taken in midlife, and 8,841 individuals had measures taken in late-life. The mean follow-up time was 9.7 years for the midlife sample, during which 8.6% developed dementia. The late-life sample had a mean follow-up time of 7.5 years, during which 24.4% developed dementia. In the STR sample, 5,856 individuals had BMI and metabolic health measures taken in midlife, out of whom 5.6% were diagnosed with dementia during a mean follow-up time of 16.6 years. BMI and metabolic health measures taken in late-life were available for 7,212 individuals, out of whom 23.1% were diagnosed with dementia during a mean follow-up time of 12.6 years.

### Risk of dementia in relation to obesity and metabolic health status measured in midlife

**Fig. 3:**
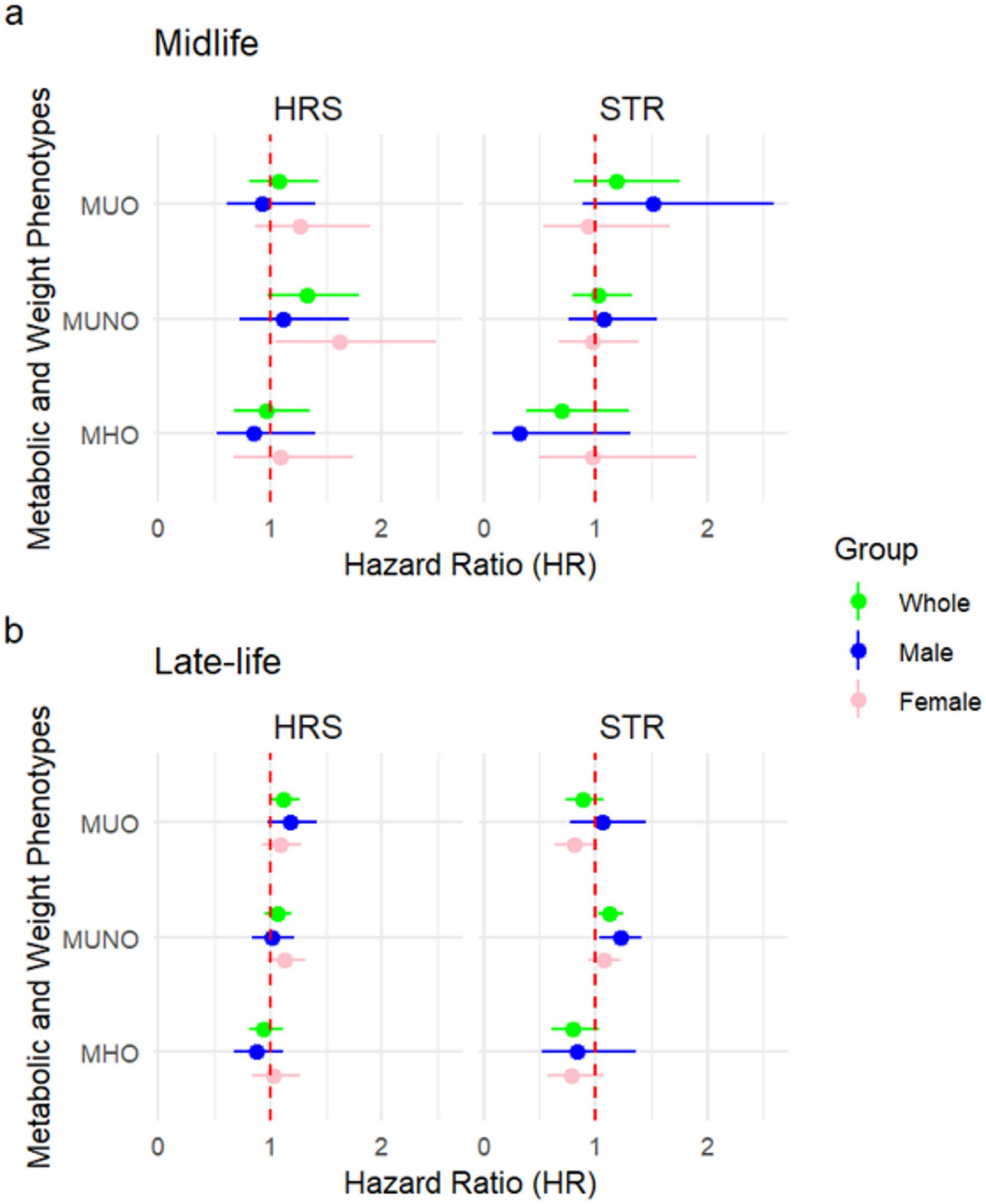
Risk of dementia in relation to obesity and metabolic health. Association between obesity and metabolic health phenotypes and dementia risk in the total analytical sample and stratified by sex, presented as hazard ratios and 95% confidence intervals. Results are based on Cox proportional hazard regression models comparing MUO, MUNO, and MHO with the reference category MHNO. All models were adjusted for age, sex, smoking status, and education level. Results are presented for measures taken at midlife (ages ≤ 65 years) and late-life (ages > 65 years), in both the HRS and STR. Abbreviations: HRS – Health and Retirement Study, MHNO – Metabolically healthy no obesity, MHO – Metabolically healthy obesity, MUNO – Metabolically unhealthy no obesity, MUO – Metabolically unhealthy obesity, STR – Swedish Twin Registry

Figure 3a presents results from the primary analyses, showing the risk of dementia in relation to obesity and metabolic health phenotypes in midlife, for the total analytical sample and stratified by sex (hazard ratio [HR] and 95% confidence interval [CI], along with the number of individuals and events for each exposure category, are presented in Additional file 1: Table S5a). None of the estimates reached statistical significance, except for MUNO in females in the HRS sample, where midlife MUNO was associated with a higher dementia risk (HR_HRS_ = 1.62 (95% CI: 1.05–2.49)). In the total sample, the HRs were indicative that being metabolically unhealthy, with or without obesity was associated with higher dementia risk compared to MHNO: (1) MUO HR_HRS_ = 1.08 (95% CI: 0.81–1.43); MUO HR_STR_ = 1.19 (95% CI 0.81–1.75); (2) MUNO HR_HRS_ = 1.33 (95% CI: 0.98–1.79); HR_STR_ = 1.03 (95% CI: 0.80–1.32). Associations were generally stronger in females than in males in the HRS sample, but not in the STR (Fig. 3a and Additional file 1: Table S5a). In the total sample, MHO indicated lower dementia risk (HR_HRS_= 0.96 (95% CI: 0.68–1.35); HR_STR_ = 0.70 (95% CI: 0.38–1.30) compared to MHNO, with a potentially stronger association in males than in females (Fig. 3a and Additional file 1: Table S5a).

Secondary analyses were in line with the primary analyses and indicated higher risk of dementia in relation to being metabolically unhealthy (HR_HRS_ = 1.19 (95% CI 0.96–1.48); HR_STR_ = 1.09 (95% CI: 0.87–1.37)), but not in relation to obesity (HR_HRS_ = 0.91 (95% CI 0.73–1.12); HR_STR_ = 0.99 (95% CI: 0.72–1.38)), with similar estimates in joint models (Additional file 1: Table S6a). In the HRS, there was no evidence of an interaction between obesity and metabolic health (HR_HRS_ interaction = 0.85 (95% CI 0.55–1.31)). In the STR, the association between being metabolically unhealthy and higher dementia risk was attenuated in the interaction model (HR_STR_ = 1.03 (95% CI: 0.80–1.32)), with a potential interaction between obesity and metabolic health (HR_STR_ interaction = 1.64 (95% CI 0.79–3.43)), especially in males (Additional file 1: Table S6a).

### Risk of dementia in relation to obesity and metabolic health measured in late-life

Figure 3b presents results from the primary analyses, showing the risk of dementia in relation to obesity and metabolic health in late-life, for the total analytical sample and stratified by sex (HR and 95% CI, along with the number of individuals and events in each exposure category, are presented in Additional file 1: Table S5b). Having MUNO in late-life was associated with a higher risk of dementia compared to MHNO, reaching statistical significance in the STR, MUNO HR_HRS_ = 1.07 (95% CI: 0.95–1.19); HR_STR_ = 1.13 (95% CI: 1.02–1.25). In sex-stratified analyses, the association was stronger in females in the HRS, but stronger in males in the STR (Fig. 3b and Additional file 1: Table S5b). Results were inconsistent for MUO, indicating a higher risk of dementia in MUO HR_HRS_ = 1.12 (95% CI: 0.99–1.26), especially in males (Fig. 3b and Additional file 1: Table S5), but lower risk in MUO HR_STR_= 0.88 (95% CI: 0.73–1.07), especially in females (Fig. 3b and Additional file 1: Table S5b), compared to MHNO. Having MHO in late-life indicated a lower dementia risk compared to MHNO, MHO HR_HRS_ = 0.94 (95% CI: 0.81–1.11); MHO HR_STR_ = 0.80 (95% CI: 0.61–1.04), although the associations were not statistically significant.

In the secondary analyses, being metabolically unhealthy was associated with higher dementia risk both in the HRS and the STR (HR_HRS_ = 1.11 (95% CI 1.01–1.21); HR_STR_ = 1.10 (95% CI: 1.00–1.22)), and the associations were robust in joint models, adjusting for obesity (Additional file 1: Table S6b). Obesity was associated with lower risk of dementia in the STR, but not in the HRS (HR_HRS_ = 1.02 (95% CI 0.94–1.12); HR_STR_ = 0.81 (95% CI: 0.69–0.94)), with comparable estimates in joint models. The main-effect estimates remained robust also in the interaction models, and there was no evidence of an interaction between obesity and metabolic health (Additional file 1: Table S6b).

**Fig. 4:**
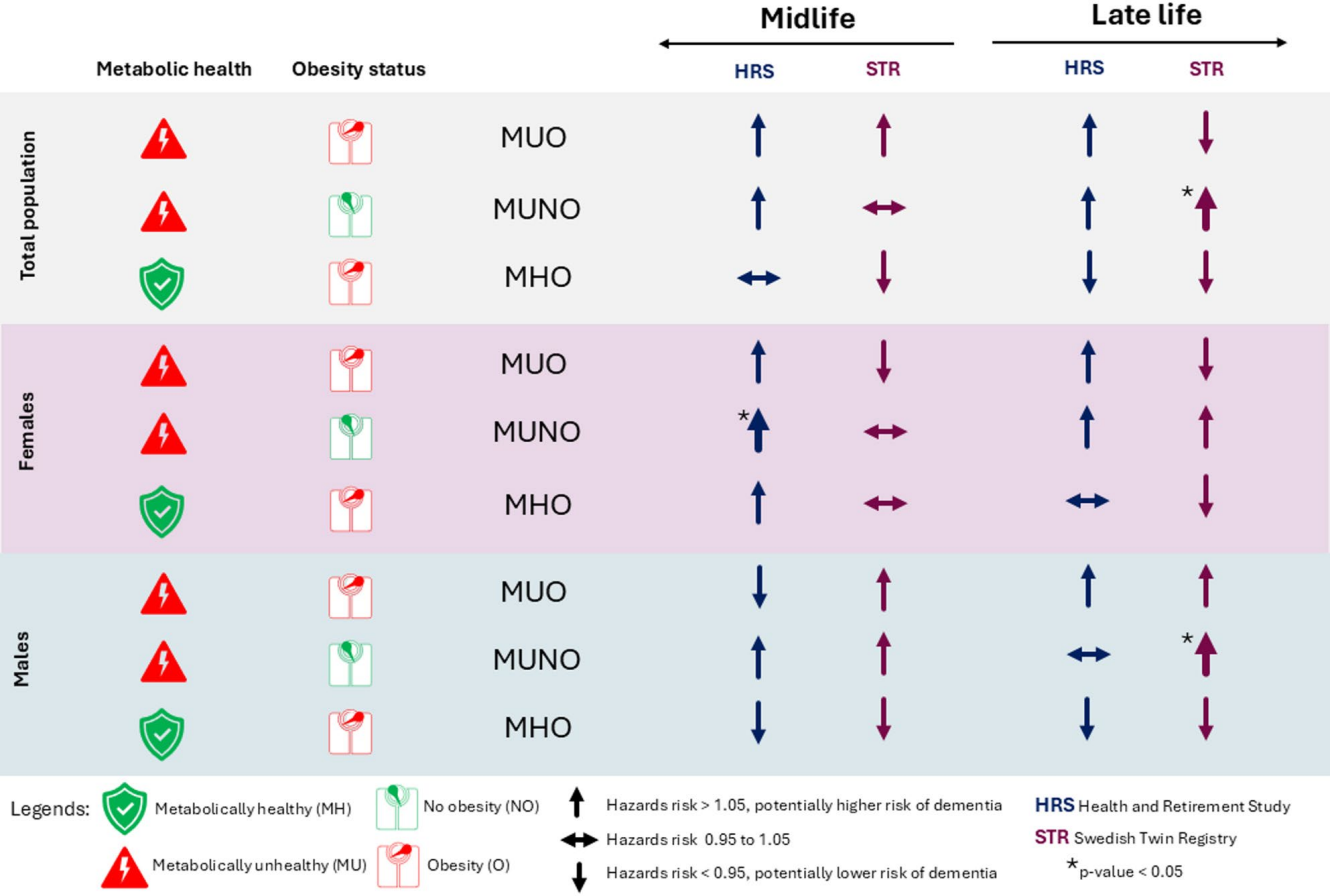
Graphical summary of the associations between obesity and metabolic health phenotypes and dementia risk. Association between obesity and metabolic health phenotypes and dementia risk in the total analytical sample and stratified by sex. Results are based on Cox proportional hazard regression models comparing MUO, MUNO, and MHO with the reference category MHNO. All models were adjusted for age, sex, smoking status, and education level. Results are presented for measures taken at midlife (ages ≤ 65 years) and late-life (ages > 65 years), in both the HRS and STR. Abbreviations: HRS – Health and Retirement Study, MHNO – Metabolically healthy no obesity, MHO – Metabolically healthy obesity, MUNO – Metabolically unhealthy no obesity, MUO – Metabolically unhealthy obesity, STR – Swedish Twin Registry

Figure 4 presents a graphical summary of the main findings.

### Sensitivity analyses

Sensitivity analyses yielded associations that were generally consistent in direction with the main analyses, although the effect sizes changed (Additional file 1: Tables S7–S14). When excluding those aged 63–67, the dementia risk was generally higher in the midlife sample, but was slightly attenuated in the late-life sample (Additional file 1: Table S7).

When metabolically unhealthy status was defined more strictly as having one or more metabolic health variables, midlife MUNO and MUO showed substantially stronger associations in the HRS, especially in the female population (Additional file 1: Table S8a). In the late-life sample, the associations were attenuated, and MUO was associated with lower dementia risk in the STR (Additional file 1: Table S8b). MHO in midlife and late-life indicated HRs in a risk-increasing direction for both HRS and STR, albeit not statistically significant (Table S8). However, it should be noted that when applying a stricter definition, only a small proportion of individuals, 16.9–22.1% in midlife and 9.2–9.9% in late-life, were categorized as being metabolically healthy. When TG was excluded from the metabolic health definition in the STR, more individuals were categorized as metabolically healthy, and associations generally attenuated for midlife measures but stronger for late-life measures of obesity and metabolic health phenotypes (Additional file 1: Table S9).

When obesity and metabolic health were classified into six categories, metabolically healthy overweight was associated with a lower dementia risk, with a statistically significant association in the HRS late-life sample. There were no clear patterns for metabolically unhealthy overweight (Additional file 1: Table S10). Across both the HRS and the STR, the overweight category was more prevalent than the normal weight category, except in midlife in the STR (Additional file 1: Table S10).

Adjusting for physical activity, alcohol consumption, additionally for depressive symptoms, and then *APOE* ε4 genotype did not change the overall pattern of associations in the midlife sample, but attenuated associations between MUNO and MUO with dementia in the HRS late-life sample (in fully adjusted model, HR_HRS_ MUNO = 0.96 (95% CI 0.86–1.08); HR_HRS_ MUO = 0.99 (95% CI 0.87–1.12); Additional file 1: Table S11 – S13).

When accounting for death as a competing event in Fine-Gray subdistribution hazards regression models, associations were comparable but slightly attenuated in the midlife sample (Additional file 1: Table S14a). In late-life, the association between MUNO and dementia was attenuated (HR_HRS_ = 1.01 (95% CI 0.92–1.12); HR_STR_ = 1.06 (95% CI: 0.95 − 1.18)), while a lower risk of dementia was seen for both MHO (HR_HRS_ = 0.78 (95% CI 0.68–0.90); HR_STR_ = 0.70 (95% CI: 0.53–0.92)), and MUO (HR_HRS_ = 0.78 (95% CI 0.71–0.87); HR_STR_ = 0.72 (95% CI: 0.59–0.88)).

## Discussion

### Principal findings

The current study examined how obesity and metabolic health together associate with dementia risk, using data from two large cohorts: the HRS and STR. In addition, we examined how these associations differ depending on the age at which obesity and metabolic health measurements were taken: midlife (≤ 65 years) or late-life (> 65 years), and by sex. In line with our hypothesis, the estimates indicated a higher dementia risk among individuals who were metabolically unhealthy in midlife, regardless of obesity status. This pattern was observed for MUO and MUNO in the primary analyses and for metabolic health status when modeled separately and jointly with obesity in secondary analyses, although the association was only statistically significant for MUNO in women in HRS in the primary analyses. Contrary to our hypothesis, having MHO in midlife was not associated with dementia risk, and obesity was not associated with dementia when modeled separately in secondary analyses.

In late-life, as hypothesized, estimates for MUNO indicated increased dementia risk, although the association reached statistical significance only in the STR. For MHO in late-life, estimates indicated a lower risk of dementia, although the results were not statistically significant in the primary analyses. In secondary analyses of the late-life sample, being metabolically unhealthy was associated with higher risk of dementia in both cohorts, while obesity was associated with lower dementia risk in the STR, but not in the HRS.

In summary, although associations were mainly not statistically significant, there was a pattern across primary and secondary analyses where being metabolically unhealthy in midlife as well as late-life was generally associated with a higher risk of dementia, regardless of weight status, although most associations were not statistically significant. In contrast, associations with MHO or obesity alone generally indicated a null association, and a possible lower risk of dementia, regardless of whether the measures were taken in midlife or late-life.

### Comparison with existing literature

Our findings are generally in line with the recent meta-analysis by Su and colleagues, based on three studies: MHO was linked to a reduced risk of dementia, whereas MUNO with an increased risk, and MUO showed no clear association [5]. Of note, the meta-analysis includes a large Korean study by Lee and colleagues, involving 12 million individuals also examining differences by age group, with a mean age of 67.1 years among those over 60 years of age, and 53.8 years among those aged 50–60 years, providing sufficient power to detect statistically significant associations even for the less prevalent phenotypes such as MHO [8]. The authors identified similar associations in individuals aged 50–60 and > 60, but with a weaker inverse association between midlife MHO and dementia risk.

By contrast, a recent study based on the Whitehall II cohort, not part of the meta-analysis by Su and colleagues, reported that individuals with MHO, MUO, and MUNO before the age of 60 showed a higher risk of dementia over a 27-year follow-up, with attenuation of associations, especially for MHO, at higher ages [9]. Similarly, a large study of three cohort studies including the UK Biobank, Atherosclerosis Risk in Communities study, and Framingham Offspring Study, with individuals having a mean age of 54–56, also showed a higher risk of dementia in individuals with MUO and MUNO [10]. For MHO, the association was in the risk-increasing direction, although smaller and not statistically significant [10]. This contrasts with our study, which indicates that MHO measured in midlife may be associated with, if anything, a lower risk of dementia. It is plausible that this difference is due to the comparatively high mean age in our current study, meaning the midlife sample largely reflects late midlife; indeed, the association between midlife MHO and dementia risk was attenuated in sensitivity analyses limiting the sample to measures taken before age 63. It should also be noted that MHO often is a temporary condition, with individuals potentially transitioning over time between metabolically healthy and unhealthy states [3, 33]. Consequently, the differing associations between MHO and dementia risk reported in previous studies may reflect variations in age at measurement, follow-up time, and these transitions in metabolic health and obesity status.

In addition, the current study was limited to all-cause dementia, as subtype diagnoses were not available in the HRS. Most previous studies have also examined all-cause dementia or Alzheimer’s disease, but Lee and colleagues additionally examined vascular dementia. Their findings indicate that obesity and metabolic health phenotypes may affect subtypes differently: MHO was associated with lower risk of Alzheimer’s disease but not with vascular dementia, and MUNO and MUO were associated with both subtypes, but associations stronger for vascular dementia than for Alzheimer’s disease [8]. While Alzheimer’s disease is the most common form of dementia, and often includes vascular pathology, subtype distribution may also contribute to differences between studies [34].

Finally, inclusion of additional covariates attenuated the associations in the late-life sample in HRS, but not the STR, and differences in lifestyle, comorbidities, and genetic background may lead to differences between studies in complex manners. For example, in a study by Shinohara and colleagues, obesity was associated with earlier cognitive decline only in non-carriers of *APOE* ε4, but at the same time, obesity was associated with lower risk of dementia, and higher cognitive ability in individuals with dementia, especially among *APOE *ε4 carriers [35]. This demonstrates complex associations between obesity, cognitive decline, and dementia, and modification from *APOE* genotype that differs between normative cognitive aging and dementia.

### Metabolic health and dementia

Taken together, the current study and previous research indicate that metabolic health is more important to dementia risk than obesity alone. Previous research has highlighted the role that metabolically unhealthy status plays in the development of dementia or cognitive impairments not due to dementia (henceforth only cognitive impairments) [36]. For instance, a recent meta-analysis including 21 studies with a total of 411,810 participants, found that the metabolic syndrome was significantly associated with increased risks of dementia and cognitive impairments [36]. As components of the metabolic syndrome are modifiable, targeted lifestyle interventions to improve metabolic health may be beneficial in preventing cognitive impairment and promoting brain health [8]. Indeed, a previous study showed that maintaining a healthy metabolic status over time represents a more influential factor of cognitive function than solely focusing on body weight [37], underscoring the importance of preserving metabolic health in dementia prevention and promoting brain health.

However, no unified definition of metabolic health in obesity exists, limiting comparability and interpretations [3, 4]. It has been suggested that MHO should require absence of all four components of the metabolic syndrome [4]. In sensitivity analyses applying this stricter definition, associations between MUNO or MUO and dementia were substantially stronger for midlife measures in the HRS but attenuated in the STR and in both samples for measures taken in late-life. In contrast, associations between MHO and dementia indicated a higher risk of dementia in MHO. While results should be interpreted with caution due to the low number of individuals defined as metabolically healthy, especially in late-life (< 10%), these differences, especially for MHO where the estimates were in different directions in primary and sensitivity analyses, demonstrate that associations are sensitive to how metabolic health is defined. This highlights a need for a better understanding of how metabolic health influences dementia and for a unified definition of MHO.

### The obesity paradox

The obesity paradox proposes that obesity in midlife is associated with an increased risk of dementia, whereas a high BMI in late-life may be associated with no increase in risk or even a reduced risk [1, 2]. For MUO, our results show a pattern consistent with obesity paradox: estimates for midlife MUO **i**ndicated a higher dementia risk, while estimates for late-life MUO were heterogeneous and associated with increased dementia risk in HRS but lower dementia risk in the STR. MHO appeared to be inversely associated with dementia risk already in midlife in the current study, although it should be noted that this may be a result of the high average age even in the midlife sample for both the HRS and STR. Considering the long preclinical phase of dementia, an inverse association between MHO and dementia may reflect prodromal dementia even in the midlife sample [34]. By late-life, estimates for MHO again indicated a lower risk of dementia compared to MHNO, a pattern suggestive of an obesity paradox.

However, sensitivity analyses with death as a competing event suggested that survival bias may play a role in the obesity paradox. In the midlife sample, the subdistribution hazards were comparable to the cause-specific hazards from the primary analyses. While this suggests that selective survival has limited influence on the estimates, it may also reflect lower impact of competing mortality at younger ages in the midlife samples. In contrast, associations between late-life MUNO and dementia were attenuated when accounting for death as a competing event, and estimates for late-life MHO and MUO indicated a lower risk of dementia. This suggests that competing mortality may partially contribute to the observed pattern, as individuals with obesity or poor metabolic health are more likely to die due to other causes before dementia is diagnosed, potentially contributing to the lower observed dementia risk. These findings suggest that selective survival may partly contribute to the obesity paradox, and that understanding the obesity paradox may require considering not only the obesity status, but also the metabolic health phenotypes, and mortality. Moreover, estimates for MUNO indicated a higher dementia risk in both midlife and late-life, although the strength of the associations varied and may have been influenced by competing mortality.

### Strengths and limitations

A major strength of this study is the use of two different large data sources with rich information and representative populations, allowing the data sources to complement each other and provide more balanced results. Another strength of our study was the use of objectively measured BMI and metabolic health in both cohorts. However, it should be noted that the populations in HRS and STR differed in characteristics; for example, the mean baseline BMI was higher in HRS, around 29–30, compared to approximately 26 in STR. The absence of data on TG and cholesterol lowering medications in the HRS restricts direct comparisons and may affect the assessment of metabolic health. Excluding TG from the metabolic health definition in the STR resulted in a larger proportion of individuals categorized as metabolically healthy, and attenuated associations in the midlife sample. While additional sample differences should be considered, the absence of TG in the HRS may have led to misclassification of participants as metabolically healthy instead of unhealthy, and resulted in attenuation of associations. This may explain the strengthening of results in sensitivity analyses of the HRS, defining a metabolically unhealthy status as having one or more of the metabolic health variables. Dementia information also differed between the cohorts, each with specific considerations. In the HRS the definition and classification of dementia was based on the Langa-Weir Classification of Cognitive Function, a validated classification and a frequently used definition in research studies [38]. However, a significant limitation to this method is that it does not capture clinical diagnosis of dementia or subtypes of dementia, but is based on cognitive measurements including tests for memory and speed of mental processing [16]. This hindered examination of subtype-specific associations, for example comparing associations with vascular dementia and Alzheimer’s disease. For the STR, clinical dementia diagnoses were not available for the TwinGene sample, but could be supplemented with information from national health registers. Dementia diagnoses from these health registers demonstrate excellent specificity (99%), although their combined sensitivity remains moderate at 63% [18]. As a result, a substantial proportion of individuals with dementia remain undetected by the registers, leading to non-differential misclassification in the outcome (assuming it is unrelated to obesity and metabolic health) and an underestimation of the true effects. While we firmly believe that the inclusion of two different cohorts, from different countries and settings, strengthens the study, these differences in exposure and outcome measures limit the comparability.

Another limitation is the relatively short follow-up time, which may have resulted in a lower ability to capture associations due to fewer incident dementia cases observed during the study period. It is also important to consider the long preclinical phase of dementia, where pathophysiological changes, such as unintentional weight loss, often begin many years before clinical onset [9, 39]. This limitation should be considered when interpreting the findings, as the timing of exposure relative to disease onset can influence the observed relationships. For example, associations may reflect prodromal changes rather than causal effects. This is relevant to the midlife as well as late-life results, considering the high mean age at baseline in the midlife sample (60 years in the HRS and 59 years in the STR). While a restricted analysis, excluding dementia incidence during the first 10 years after measurement, would be preferable, the limited follow-up would have led to substantially reduced power in the current study. Furthermore, as with many studies of late-life outcomes, survival bias and attrition rate must be considered. Those with poorer health are often underrepresented in the study sample [40], and a risk that should be considered is that individuals living with obesity or with poor metabolic health may be less likely to participate or to drop out of the study due to poor health or death, which would limit our ability to capture how obesity and metabolic health associates with dementia risk. As seen in competing risk sensitivity analyses, survival bias likely influences associations, especially in the late-life sample where mortality was more common. Finally, while the two cohorts are valuable for within-twin pair (STR) and household (HRS) analyses, we did not include such analyses in the current study, as the lower power would have limited comparability with the primary analyses. Future work may consider within-twin and household models to specifically assess the influence of unmeasured genetic and environmental confounding in associations between obesity, metabolic health, and dementia.

## Conclusions

Leveraging data from the HRS and STR in a prospective cohort design, our findings suggest that metabolically unhealthy status may be associated with an increased risk of dementia regardless of obesity status, although statistical significance was only observed for selected associations. MUNO showed the clearest pattern of higher dementia risk when assessed in midlife and late-life, while the associations for MHO were more heterogeneous, potentially reflecting the obesity paradox. In contrast, MHO was not associated with higher dementia risk in either age group, with estimates indicating a possible lower dementia risk compared with metabolically healthy no obesity, although these associations were not statistically significant.

In the context of healthy aging, our findings indicate an importance of metabolic health as a key dimension of dementia risk, not only among individuals with obesity but also in the absence of obesity. These results support a more nuanced approach to risk stratification, highlighting metabolic dysfunction as a potential target for screening and interventions aimed at preserving cognitive function across the aging process. By examining differential associations across metabolic health, obesity status, age at assessment, and sex, this study strengthens the understanding of how obesity and metabolic health interact in relation to cognitive aging, with potential relevance for population-level efforts and prevention strategies to support healthy aging.

## Supplementary Information

Below is the link to the electronic supplementary material.

Supplementary Material 1: Additional file 1: Table S1 – ICD-codes for dementia; Table S2 – ATC-codes for dementia medication identification; Table S3 – Diagnostic criteria for the metabolic health variables; Table S4. Creation of physical activity measures; Table S5 – Dementia risk by obesity-metabolic health phenotypes; Table S6 – Separate, joint, and interaction models; Table S7 – Dementia risk in age-specific exposure definitions; Table S8 – Dementia risk with alternative metabolic health definition; Table S9 – Dementia risk excluding triglycerides in metabolic health definition; Table S10 – Dementia risk in six obesity-metabolic health phenotypes; Table S11 – Additionally adjusting for physical activity and alcohol consumption; Table S12 – Additionally adjusting for depressive symptoms, physical activity, and alcohol consumption; Table S13 – Additionally adjusting for depressive symptoms, physical activity, alcohol consumption, and Apolipoprotein E ε4; Table S14 – Competing risk regression modelling with death as the competing event

Supplementary Material 2

## Data Availability

The data involved in this study were obtained from the Health and Retirement Study (HRS) and the Swedish Twin Registry (STR). STR is an international resource that can be applied for access at: https://ki.se/en/research/swedish-twin-registry-for-researchers. The Health and Retirement Study data are produced and distributed by the University of Michigan with funding from the National Institute on Aging (grant number NIA U01AG009740), Ann Arbor, MI. The public use data are available through registration at the HRS website: https://hrs.isr.umich.edu/. Additional registration is required for access to sensitive biomarker and health data. The current study used the Langa-Weir Classification of Cognitive Function (1995 - 2020), May 2023, and biomarker data from the waves in 2006 (https://doi.org/10.7826/UMWV1829), 2008 (https://doi.org/10.7826/URPP9667), 2010 (https://doi.org/10.7826/ANHP6186), 2012 (https://doi.org/10.7826/UVTJ1509), 2014 (https://doi.org/10.7826/AJRT7105), and 2016 (https://doi.org/10.7826/GDSJ6138). In addition, we used the RAND HRS Longitudinal File 2022 (V1), produced by the RAND Center for the Study of Aging with funding from the National Institute on Aging and the Social Security Administration, Santa Monica, CA.

## Abbreviations

*APOE*: Apolipoprotein E
BMI: Body mass index
BP: Blood pressure
CDR: Cause of Death Register
CI: Confidence interval
CIND: Cognitively Impaired but no Dementia
GED: General Educational Development
GENDER: Aging in Women and Men
HbA1c: Hemoglobin A1c
HDL-C: High–density lipoprotein cholesterol
HR: Hazard ratio
HRS: Health and Retirement Study
MHNO: Metabolically healthy no obesity
MHO: Metabolically healthy obesity
MUNO: Metabolically unhealthy no obesity
MUO: Metabolically unhealthy obesity
N: Number of individuals
NPR: National Patient Register
OCTO-Twin: Origins of Variance in the Oldest Old: Octogenarian Twins
SATSA: The Swedish Adoption/Twin Study of Aging
SD: Standard deviation
STR: Swedish Twin Registry
TG: Triglycerides
