# Supplementary material for: Dementia risk by metabolic health and obesity in two prospective cohorts
Source: BMC Med. 2026 Jun 19;24:366. doi: 10.1186/s12916-026-05002-8 (PMC13292518; doi:10.1186/s12916-026-05002-8)
Supplement: Supplementary file 1 — Supplementary Material 1: Additional file 1: Table S1 – ICD-codes for dementia; Table S2 – ATC-codes for dementia medication identification; Table S3 – Diagnostic criteria for the metabolic health variables; Table S4. Creation of physical activity measures; Table S5 – Dementia risk by obesity-metabolic health phenotypes; Table S6 – Separate, joint, and interaction models; Table S7 – Dementia risk in age-specific exposure definitions; Table S8 – Dementia risk with alternative metabolic health definition; Table S9 – Dementia risk excluding triglycerides in metabolic health definition; Table S10 – Dementia risk in six obesity-metabolic health phenotypes; Table S11 – Additionally adjusting for physical activity and alcohol consumption; Table S12 – Additionally adjusting for depressive symptoms, physical activity, and alcohol consumption; Table S13 – Additionally adjusting for depressive symptoms, physical activity, alcohol consumption, and Apolipoprotein E ε4; Table S14 – Competing risk regression modelling with death as the competing event [file 12916_2026_5002_MOESM1_ESM.pdf]

Additional file 1

Dementia Risk by Metabolic Health and Obesity in Two Prospective Cohorts

Martin Nakash, Elsa Ojalehto Lindfors, Yiqiang Zhan, Anna K. Dahl Aslan, Chandra A. Reynolds, Peggy Ler, Ida K. Karlsson

Contents

Table S1. ICD-codes used to identify dementia in the Swedish Twin Registry. 1

Table S2. ATC-codes for identification of dementia medication in the Swedish Twin Registry. 2

Table S3. Diagnostic criteria for the metabolic health variables. 3

Table S4. Creation of physical activity measure in the Swedish Twin Registry studies. 3

Table S5. Risk of dementia in relation to obesity and metabolic health phenotypes. 4

Table S6. Separate, joint, and interaction models of risk of dementia in relation to obesity and poor metabolic health 5

Table S7. Risk of dementia in relation to obesity and metabolic health phenotypes measured at ages <63 years for midlife and ages >67 for late life. 7

Table S8. Risk of dementia in relation to obesity and metabolic health, where metabolically unhealthy status was defined as having at least one unhealthy metabolic component. 8

Table S9. Risk of dementia in relation to obesity and metabolic health in the STR, where metabolically unhealthy status was defined without triglycerides (based on hypertension, hyperglycemia, and dyslipidemia (HDL-C)) 9

Table S10. Risk of dementia in relation to obesity and metabolic health phenotypes, classified as six categories. 11

Table S11. Risk of dementia in relation to obesity and metabolic health phenotypes, additionally adjusting for physical activity and alcohol consumption. 12

Table S12. Risk of dementia in relation to obesity and metabolic health phenotypes, additionally adjusting for depressive symptoms, physical activity, and alcohol consumption. 13

Table S13. Risk of dementia in relation to obesity and metabolic health phenotypes, additionally adjusting for depressive symptoms, physical activity, alcohol consumption, and Apolipoprotein E ε4. 15

Table S14. Risk of dementia in relation to obesity and metabolic health phenotypes, from competing risk regression modelling death as the competing event 16

Table S1. ICD-codes used to identify dementia in the Swedish Twin Registry.

ICD-7	ICD-8	ICD-9*	ICD-10
**(used before 1969)**	**(used 1969-1986)**	**(used 1987-1996)**	**(used 1997 and onwards)**
**304** Senile psychosis	**290** Senile and presenile dementia	**290** Senile and presenile organic psychotic condition	**F00** Dementia in Alzheimer's disease
**305** Presenile psychosis	**293.0** Cerebral arteriosclerosis	**294B/ 294.1** Dementia in conditions classified elsewhere	**F01** Vascular dementia
**306** Psychosis with cerebral arteriosclerosis	**293.1** Other cerebrovascular disturbances	**331A/ 331.0** Alzheimer's disease	**F02** Dementia in other diseases classified elsewhere
		**331B/ 331.1** Pick's disease	**F03** Unspecified dementia
		**331C/ 331.2** Senile degeneration of brain	**F051** Delirium superimposed on dementia
		**331X/ 331.9** Cerebral degeneration, unspecified	**G30** Alzheimer's disease
			**G311** Senile degeneration of brain, not elsewhere classified
			**G318A** Other specified degenerative diseases of nervous system: Lewy body dementia

ICD-codes used to identify dementia diagnosis. By using the unique personal identification number assigned to all Swedish residents, diagnoses were obtained from the National Patient Register (NPR) and the Cause of Death Register (CDR) [18].

* In the Swedish adaptation of ICD-9, the 4th digit was replaced with a letter. While the Swedish National Patient Register used the Swedish adaptation of ICD-9, the ICD versions was used in Cause of Death Registries, therefore, both versions of the codes are reported here.

Table S2. ATC-codes for identification of dementia medication in the Swedish Twin Registry.

**N06DA** Anticholinesterases
N06DA02 Donepezil<br>N06DA03 Rivastigmine<br>N06DA04 Galantamine<br>(N06DA01 Tacrine and N06DA05 Ipidacrine not prescribed in Sweden)
**N06DX** Other anti-dementia drugs
N06DX01 Memantine<br>(N06DX02 Ginkgo folium not prescribed in Sweden)

The ATC-codes for dementia medication. Prescribed dementia medication from the Prescribed Drug Register was used as a proxy to the diagnoses [17].

Table S3. Diagnostic criteria for the metabolic health variables.

**Metabolic health variables**	**Diagnostic criteria**	
	**The HRS**	**The STR**
**Hypertension**	SBP ≥130 mmHg or	SBP ≥130 mmHg or
	DBP ≥85 mmHg	DBP ≥85 mmHg
	Self-reported hypertension	Self-reported hypertension
**Hyperglycemia**	HbA1c >5.7% or	HbA1c >5.7% or
	Self-reported diabetes	Self-reported diabetes or
		Fasting BG ≥6.1 mmol/L or
		Non-fasting BG ≥7.0 mmol/L
**Dyslipidemia (TG)**	No data	Fasting TG ≥1.7 mmol/ or
		Non-fasting TG ≥2.1 mmol/L
		Use of cholesterol lowering medication
**Dyslipidemia (HDL-C)**	HDL-C <40 mg/dL in males or	HDL-C <1.03 mmol/L in males or
	HDL-C <50 mg/dL in females	HDL-C <1.30 in females
		Use of cholesterol lowering medication.
**Metabolically unhealthy status**	≥2 of 3 metabolic health variables	≥2 of 4 metabolic health variables

The diagnostic criteria for hypertension, hyperglycemia, and dyslipidemia used in the HRS and the STR.

Abbreviations. BG – Blood glucose. DBP – Diastolic blood pressure. HbA1c – Hemoglobin A1c. HDL-C – High-Density Lipoprotein Cholesterol. HRS – Health and Retirement Study. SBP – Systolic blood pressure. STR – Swedish Twin Registry. TG – Triglycerides.

Table S4. Creation of physical activity measure in the Swedish Twin Registry studies.

Physical activity	SALT: How much do you exercise?	SALT: Of these 7 alternatives, which fits your annual exercise pattern?	SATSA: Please state below how much exercise you get during your leisure time.	GENDER: Leisure activities - do you exercise	GENDER: Leisure activities - do you walk
0	0: Almost no exercise	0: Almost never exercise<br>1: Much less exercise than average<br>2: Less exercise than average	1: I hardly get any exercise at all<br>2: I get very little exercise	5: Never	5: Never<br>4: more seldom than once/week<br>3: once or a few/month
1	1: Light exercise, e.g., regular promenades or light garden walk	3: Average amount of exercise	3: I get little exercise<br>4: I don't get very much exercise	4: more seldom than once/week<br>3: once or a few/month	2: once or a few/week<br>1: daily
2	2: Regular medium exercise<br>3: Hard physical exercise	4: More exercise than average<br>5: Much more exercise than average<br>6: Maximum amount of exercise	5: I get quite a lot of exercise<br>6: I get a lot of exercise<br>7: I get very much exercise	2: once or a few/week<br>1: daily	

A new three-level variable for physical activity was created, based on different questions from the sub-studies from the Swedish Twin Registry. In SALT, participants born 1925 or earlier were asked how much they exercise (first column), and participants born after 1925 were asked about their annual exercise pattern (second column). In SATSA, the first, second, third, seventh, eighth, and ninth questionnaire asked about leisure time exercise (third column). The response closest in age to the biomarker measure was used. In GENDER, the first questionnaire included questions on leisure time exercise and walking.

Table S5. Risk of dementia in relation to obesity and metabolic health phenotypes.

a) Midlife	The HRS				The STR			
	N	Events	HR	95% CI	N	Events	HR	95% CI
**Total analytical sample**	4,233	362			5,856	327		
MHNO	1,480	86	Ref	Ref	3,404	192	Ref	Ref
MHO	839	57	0.96	0.68 – 1.35	298	11	0.70	0.38 – 1.30
MUNO	726	90	1.33	0.98 – 1.79	1,622	94	1.03	0.80 – 1.32
MUO	1,188	129	1.08	0.81 – 1.43	532	30	1.19	0.81 – 1.75
**Male sample**	1,715	161			2,590	146		
MHNO	602	47	Ref	Ref	1,314	73	Ref	Ref
MHO	353	24	0.85	0.52 – 1.40	105	2	0.32	0.08 – 1.31
MUNO	346	42	1.12	0.73 – 1.71	905	54	1.08	0.76 – 1.55
MUO	414	48	0.93	0.61 – 1.41	266	17	1.52	0.88 – 2.60
**Female sample**	2,518	201			3,266	181		
MHNO	878	39	Ref	Ref	2,090	119	Ref	Ref
MHO	486	33	1.09	0.68 – 1.75	193	9	0.97	0.49 – 1.91
MUNO	380	48	**1.62**	**1.05 – 2.49**	717	40	0.97	0.67 – 1.39
MUO	774	81	1.27	0.86 – 1.89	266	13	0.93	0.53 – 1.66

b) Late-life	The HRS				The STR			
	N	Events	HR	95% CI	N	Events	HR	95% CI
**Total analytical sample**	8,841	2,078			7,212	1,669		
MHNO	2,702	586	Ref	Ref	3,483	806	Ref	Ref
MHO	1,134	213	0.94	0.81 – 1.11	318	59	0.80	0.61 – 1.04
MUNO	2,463	700	1.07	0.95 – 1.19	2,745	680	**1.13**	**1.02 – 1.25**
MUO	2,542	579	1.12	0.99 – 1.26	666	124	0.88	0.73 – 1.07
**Male sample**	3,759	847			3,349	710		
MHNO	1,138	250	Ref	Ref	1,511	314	Ref	Ref
MHO	505	88	0.88	0.68 – 1.12	106	17	0.84	0.52 – 1.37
MUNO	1,058	271	1.01	0.84 – 1.21	1,459	333	**1.22**	**1.04 – 1.42**
MUO	1,058	238	1.18	0.98 – 1.42	273	46	1.06	0.77 – 1.45
**Female sample**	5,082	1,231			3,863	959		
MHNO	1,564	336	Ref	Ref	1,972	492	Ref	Ref
MHO	629	125	1.03	0.84 – 1.27	212	42	0.78	0.57 – 1.07
MUNO	1,405	429	1.13	0.98 – 1.31	1,286	347	1.07	0.93 – 1.23
MUO	1,484	341	1.09	0.93 – 1.28	393	78	0.81	0.63 – 1.03

Hazard rate ratios (HR) and 95% confidence intervals (CI) from Cox proportional hazard regression, showing risk of dementia in relation to MUO, MUNO, and MHO compared to the reference category MHNO. Results are presented for measures taken in a) midlife (age ≤65 years) late-life (>65 years), in the total analytical sample, the male analytical sample, and the female analytical sample. All models were adjusted for age, sex, smoking status, and education level. Models of the HRS were further adjusted for ethnicity, and models of the STR for sub-study. Bold numbers indicate statistical significance at the α=0.05 level.

Abbreviations: CI – Confidence interval, HR – Hazard ratio, HRS – Health and Retirement Study, N – Number of individuals, MHNO – Metabolically healthy no obesity, MHO – Metabolically healthy obesity, MUNO – Metabolically unhealthy no obesity, MUO – Metabolically unhealthy obesity, STR – Swedish Twin Registry.

Table S6. Separate, joint, and interaction models of risk of dementia in relation to obesity and poor metabolic health

a) Midlife		The HRS		The STR	
**Total analytical sample**		**HR**	**95% CI**	**HR**	**95% CI**
Model 1: Obesity	Obesity	0.91	0.73 – 1.12	0.99	0.72 – 1.38
Model 2: MU	MU	1.19	0.96 – 1.48	1.09	0.87 – 1.37
Model 3: Joint model, obesity and MU	Obesity	0.87	0.70 – 1.08	0.97	0.69 – 1.36
	MU	1.23	0.98 – 1.53	1.09	0.86 – 1.38
Model 4: Interaction model, obesity and MU	Obesity	0.96	0.68 – 1.35	0.70	0.38 – 1.30
	MU	1.33	0.98 – 1.79	1.03	0.80 – 1.32
	Obesity*MU	0.85	0.55 – 1.31	1.64	0.79 – 3.43
**Male sample**					
Model 1: Obesity	Obesity	0.86	0.62 – 1.18	1.05	0.64 – 1.72
Model 2: MU	MU	1.07	0.78 – 1.48	1.23	0.88 – 1.71
Model 3: Joint model, obesity and MU	Obesity	0.84	0.61 – 1.16	1.01	0.61 – 1.65
	MU	1.11	0.80 – 1.54	1.23	0.88 – 1.72
Model 4: Interaction model, obesity and MU	Obesity	0.85	0.52 – 1.40	0.32	0.08 – 1.31
	MU	1.12	0.73 – 1.71	1.08	0.76 – 1.55
	Obesity*MU	0.97	0.51 – 1.87	4.36	0.96 – 19.7
**Female sample**					
Model 1: Obesity	Obesity	0.95	0.71 – 1.27	0.96	0.61 – 1.50
Model 2: MU	MU	1.33	0.99 – 1.80	0.96	0.69 – 1.33
Model 3: Joint model, obesity and MU	Obesity	0.89	0.66 – 1.19	0.97	0.61 – 1.53
	MU	**1.37**	**1.01 – 1.86**	0.97	0.69 – 1.35
Model 4: Interaction model, obesity and MU	Obesity	1.09	0.68 – 1.75	0.97	0.49 – 1.91
	MU	**1.62**	**1.05 – 2.49**	0.97	0.67 – 1.39
	Obesity*MU	0.72	0.40 – 1.30	1.00	0.40 – 2.52

b) Late-life		The HRS		The STR	
**Total analytical sample**		**HR**	**95% CI**	**HR**	**95% CI**
Model 1: Obesity	Obesity	1.02	0.94 – 1.12	**0.81**	**0.69 – 0.94**
Model 2: MU	MU	**1.11**	**1.01 – 1.21**	**1.10**	**1.00 – 1.22**
Model 3: Joint model, obesity and MU	Obesity	1.01	0.92 – 1.11	**0.79**	**0.67 – 0.92**
	MU	**1.10**	**1.01 – 1.21**	**1.13**	**1.02 – 1.25**
Model 4: Interaction model, obesity and MU	Obesity	0.94	0.81 – 1.11	0.80	0.61 – 1.04
	MU	1.07	0.95 – 1.19	**1.13**	**1.02 – 1.26**
	Obesity*MU	1.11	0.91 – 1.34	0.98	0.71 – 1.36
**Male sample**					
Model 1: Obesity	Obesity	1.07	0.93 – 1.23	0.89	0.69 – 1.16
Model 2: MU	MU	1.12	0.97 – 1.30	**1.21**	**1.04 – 1.40**
Model 3: Joint model, obesity and MU	Obesity	1.05	0.91 – 1.22	0.86	0.66 – 1.12
	MU	1.11	0.96 – 1.29	**1.22**	**1.05 – 1.42**
Model 4: Interaction model, obesity and MU	Obesity	0.88	0.68 – 1.12	0.84	0.52 – 1.37
	MU	1.01	0.84 – 1.21	**1.22**	**1.04 – 1.43**
	Obesity*MU	1.33	0.98 – 1.80	1.03	0.58 – 1.84

Female sample					
Model 1: Obesity	Obesity	1.00	0.89 – 1.13	**0.77**	**0.64 – 0.94**
Model 2: MU	MU	1.10	0.98 – 1.24	1.04	0.91 – 1.18
Model 3: Joint model, obesity and MU	Obesity	0.99	0.87 – 1.12	**0.76**	**0.63 – 0.92**
	MU	1.11	0.98 – 1.25	1.07	0.94 – 1.22
Model 4: Interaction model, obesity and MU	Obesity	1.03	0.84 – 1.27	0.78	0.57 – 1.07
	MU	1.13	0.98 – 1.31	1.07	0.93 – 1.23
	Obesity*MU	0.94	0.73 – 1.21	0.97	0.65 – 1.45

Hazard rate ratios (HR) and 95% confidence intervals (CI) from Cox proportional hazard regression modelling 1) obesity separately, 2) metabolically unhealthy status (MU) separately, 3) obesity and MU jointly, and 4) an interaction between obesity and MU. Results are presented for measures taken in a) midlife (≤65 years) and b) late-life (>65 years), in the HRS and the STR. All models were adjusted for age, sex, smoking status, and education level. Models of the HRS were further adjusted for ethnicity, and models of the STR for sub-study. Bold numbers indicate statistical significance at the α=0.05 level.

Abbreviations: CI – Confidence interval, HR – Hazard ratio, HRS – Health and Retirement Study, MU – Metabolically unhealthy, STR – Swedish Twin Registry.

Table S7. Risk of dementia in relation to obesity and metabolic health phenotypes measured at ages <63 years for midlife and ages >67 for late life.

a) Midlife	The HRS				The STR			
	N	Events	HR	95% CI	N	Events	HR	95% CI
**Total analytical sample**	3,356	248			4,545	222		
MHNO	1,208	59	Ref	Ref	2,699	127	Ref	Ref
MHO	696	42	1.12	0.75 – 1.68	230	9	0.89	0.45 – 1.76
MUNO	543	57	1.20	0.83 – 1.74	1,202	64	1.27	0.93 – 1.74
MUO	909	90	1.31	0.93 – 1.84	414	22	1.29	0.82 – 2.04
**Male sample**	1,372	114			2,002	92		
MHNO	483	31	Ref	Ref	1,028	38	Ref	Ref
MHO	300	19	1.08	0.61 – 1.94	76	2	0.68	0.16 – 2.84
MUNO	268	29	1.00	0.59 – 1.94	690	40	**1.80**	**1.14 – 2.84**
MUO	321	35	1.16	0.71 – 1.91	208	12	**2.04**	**1.04 – 3.99**
**Female sample**	1,984	134			2,543	130		
MHNO	725	28	Ref	Ref	1,671	89	Ref	Ref
MHO	396	23	1.23	0.70 – 2.16	154	7	1.01	0.47 – 2.19
MUNO	275	28	1.48	0.86 – 2.55	512	24	0.95	0.60 – 1.50
MUO	588	55	1.54	0.96 – 2.48	206	10	0.95	0.49 – 1.82

b) Late-life	The HRS				The STR			
	N	Events	HR	95% CI	N	Events	HR	95% CI
**Total analytical sample**	6,467	1,827			5,257	1,405		
MHNO	2,034	532	Ref	Ref	2,499	686	Ref	Ref
MHO	796	187	0.94	0.80 – 1.11	242	50	0.76	0.57 – 1.01
MUNO	1,890	624	1.04	0.93 –1.17	2,044	570	1.09	0.97 – 1.22
MUO	1,747	484	1.08	0.96 – 1.23	472	99	0.83	0.67 – 1.03
**Male sample**	2,793	746			2,460	597		
MHNO	874	225	Ref	Ref	1,105	272	Ref	Ref
MHO	373	80	0.85	0.66 – 1.10	78	14	0.77	0.45 – 1.32
MUNO	803	240	0.97	0.80 – 1.16	1,086	277	1.15	0.97 – 1.37
MUO	743	201	1.13	0.93 – 1.37	191	34	0.93	0.65 – 1.33
**Female sample**	3,674	1,081			2,797	808		
MHNO	1,160	307	Ref	Ref	1,394	414	Ref	Ref
MHO	423	107	1.05	0.84 – 1.30	164	36	0.75	0.53 – 1.06
MUNO	1,087	384	1.10	0.95 – 1.28	958	293	1.05	0.90 – 1.22
MUO	1,004	283	1.06	0.90 – 1.26	281	65	0.79	0.61 – 1.03

Hazard rate ratios (HR) and 95% confidence intervals (CI) from Cox proportional hazard regression, showing risk of dementia in relation to MUO, MUNO, and MHO compared to the reference category MHNO. Results are presented for measures taken at a) ages <63 for midlife, and b) ages >67 years for late life, in the total analytical sample, the male analytical sample, and the female analytical sample. All models were adjusted for age, sex, smoking status, and education level. Models of the HRS were further adjusted for ethnicity, and models of the STR for sub-study. Bold numbers indicate statistical significance at the α=0.05 level.

Abbreviations: CI – Confidence interval, HR – Hazard ratio, HRS – Health and Retirement Study, N – Number of individuals, MHNO – Metabolically healthy no obesity, MHO – Metabolically healthy obesity, MUNO – Metabolically unhealthy no obesity, MUO – Metabolically unhealthy obesity, STR – Swedish Twin Registry.

Table S8. Risk of dementia in relation to obesity and metabolic health, where metabolically unhealthy status was defined as having at least one unhealthy metabolic component.

a) Midlife	The HRS				The STR			
	N	Events	HR	95% CI	N	Events	HR	95% CI
**Total analytical sample**	4,233	362			5,856	327		
MHNO	547	13	Ref	Ref	1,245	56	Ref	Ref
MHO	168	8	1.76	0.73 –4.25	51	3	1.57	0.49 – 5.03
MUNO	1,659	163	**2.35**	**1.33 – 4.15**	3,781	230	0.94	0.70 –1.26
MUO	1,859	178	**1.96**	**1.11 – 3.47**	779	38	0.92	0.60 – 1.38
**Male sample**	1,715	161			2,590	146		
MHNO	189	7	Ref	Ref	436	25	Ref	Ref
MHO	59	3	1.22	0.32 – 4.76	13	1	1.06	0.14 – 7.90
MUNO	759	82	1.88	0.86 – 4.11	1,783	102	0.76	0.49 – 1.18
MUO	708	69	1.53	0.69 – 3.37	358	18	0.83	0.45 – 1.53
**Female sample**	2,518	201			3,266	181		
MHNO	358	6	Ref	Ref	809	31	Ref	Ref
MHO	109	5	2.33	0.71 – 7.68	38	2	1.95	0.47 – 8.17
MUNO	900	81	**2.95**	**1.28 – 6.82**	1,998	128	1.10	0.74 – 1.63
MUO	1,151	109	**2.51**	**1.09 – 5.80**	421	20	0.98	0.56 – 1.73

b) Late-life	The HRS				The STR			
	N	Events	HR	95% CI	N	Events	HR	95% CI
**Total analytical sample**	8,841	2,078			7,212	1,669		
MHNO	655	118	Ref	Ref	684	155	Ref	Ref
MHO	156	28	0.99	0.65 – 1.50	28	10	1.84	0.97 – 3.50
MUNO	4,510	1,168	0.98	0.81 – 1.19	5,544	1,331	0.98	0.83 –1.16
MUO	3,520	764	1.01	0.83 – 1.23	956	173	**0.77**	**0.62 – 0.96**
**Male sample**	3,759	847			3,349	710		
MHNO	248	50	Ref	Ref	302	68	Ref	Ref
MHO	65	13	0.95	0.51 – 1.76	7	2	2.11	0.52 – 8.63
MUNO	1,948	471	0.91	0.68 – 1.23	2,668	579	0.99	0.77 – 1.27
MUO	1,498	313	0.99	0.73 – 1.34	372	61	0.87	0.61 – 1.23
**Female sample**	5,082	1,231			3,863	959		
MHNO	407	68	Ref	Ref	382	87	Ref	Ref
MHO	91	15	1.01	0.57 – 1.77	21	8	1.71	0.83 – 3.53
MUNO	2,562	697	1.05	0.81 – 1.35	2,876	752	0.98	0.78 – 1.22
MUO	2,022	451	1.05	0.81 – 1.36	584	112	**0.73**	**0.55 – 0.97**

Hazard rate ratios (HR) and 95% confidence intervals (CI) from Cox proportional hazard regression, showing risk of dementia in relation to MUO, MUNO, and MHO compared to the reference category MHNO. Metabolically healthy status was defined as having no hypertension, hyperglycemia or dyslipidemia. Results are presented for measures taken in a) midlife (≤65 years) and b) late-life (>65 years), in the total analytical sample, the male analytical sample, and the female analytical sample. All models were adjusted for age, sex, smoking status, and education level. Models of the HRS were further adjusted for ethnicity, and models of the STR for sub-study. Bold numbers indicate statistical significance at the α=0.05 level.

Abbreviations: CI – Confidence interval, HR – Hazard ratio, HRS – Health and Retirement Study, N – Number of individuals, MHNO – Metabolically healthy no obesity, MHO – Metabolically healthy obesity, MUNO – Metabolically unhealthy no obesity, MUO – Metabolically unhealthy obesity, STR – Swedish Twin Registry.

Table S9. Risk of dementia in relation to obesity and metabolic health in the STR, where metabolically unhealthy status was defined without triglycerides (based on hypertension, hyperglycemia, and dyslipidemia (HDL-C))

	a) Midlife				b) Late-life			
	**N**	**Events**	**HR**	**95% CI**	**N**	**Events**	**HR**	**95% CI**
**Total analytical sample**	5,856	327			7,228	1,671		
MHNO	3,872	219	Ref	Ref	4,252	983	Ref	Ref
MHO	417	21	0.99	0.63 – 1.55	468	91	0.83	0.67 – 1.03
MUNO	1,154	67	1.02	0.77 – 1.34	1,989	505	**1.22**	**1.10 – 1.37**
MUO	413	20	1.01	0.64 – 1.60	519	92	0.89	0.72 – 1.10
**Male sample**	2,590	146			3,356	710		
MHNO	1,594	89	Ref	Ref	1,881	391	Ref	Ref
MHO	155	6	0.73	0.32 – 1.68	171	33	1.02	0.71 – 1.47
MUNO	625	38	1.10	0.75 – 1.62	1,093	256	**1.29**	**1.10 – 1.51**
MUO	216	13	1.39	0.77 – 2.52	211	30	0.94	0.64 – 1.36
**Female sample**	3,266	181			3,872	961		
MHNO	2,278	130	Ref	Ref	2,371	592	Ref	Ref
MHO	262	15	1.16	0.68 – 1.98	297	58	**0.75**	**0.57 – 0.98**
MUNO	529	29	0.94	0.62 – 1.40	896	249	**1.18**	**1.01 – 1.37**
MUO	197	7	0.68	0.31 – 1.45	308	62	0.88	0.67 –1.14

Hazard rate ratios (HR) and 95% confidence intervals (CI) from Cox proportional hazard regression, showing risk of dementia in relation to MUO, MUNO, and MHO compared to the reference category MHNO. Metabolically healthy status was defined as having maximum one metabolic component out of hypertension, hyperglycemia, or dyslipidemia based on HDL-C (excluding triglycerides). Results are presented for measures taken in a) midlife (≤65 years) and b) late-life (>65 years), in the total analytical sample, the male analytical sample, and the female analytical sample. All models were adjusted for age, sex, smoking status, education level, and sub-study. Bold numbers indicate statistical significance at the α=0.05 level.

Abbreviations: CI – Confidence interval, HR – Hazard ratio, N – Number of individuals, MHNO – Metabolically healthy no obesity, MHO – Metabolically healthy obesity, MUNO – Metabolically unhealthy no obesity, MUO – Metabolically unhealthy obesity, TG – Triglycerides.

Table S10. Risk of dementia in relation to obesity and metabolic health phenotypes, classified as six categories.

a) Midlife	The HRS				The STR			
	N	Events	HR	95% CI	N	Events	HR	95% CI
**Total analytical sample**	4,233	362			5,856	327		
MHNW	579	36	Ref	Ref	2,050	112	Ref	Ref
MHOW	901	50	0.82	0.53 – 1.26	1,354	80	1.09	0.82 – 1.45
MHO	839	57	0.85	0.56 – 1.30	298	11	0.73	0.39 – 1.36
MUNW	177	24	1.08	0.64 –1.83	582	40	1.29	0.89 – 1.86
MUOW	549	66	1.21	0.81 –1.83	1,040	54	0.94	0.67 – 1.31
MUO	1188	129	0.96	0.66 – 1.40	532	30	1.23	0.82 – 1.85
**Male sample**	1,715	161			2,590	146		
MHNW	175	18	Ref	Ref	689	45	Ref	Ref
MHOW	427	29	1.00	0.55 –1.82	625	28	0.71	0.44 – 1.15
MHO	353	24	0.86	0.46 – 1.60	105	2	0.28	0.07 – 1.15
MUNW	76	11	1.03	0.48 – 2.20	246	17	1.24	0.71 – 2.18
MUOW	270	31	1.16	0.64 – 2.09	659	37	0.84	0.54 – 1.31
MUO	414	48	0.93	0.54 – 1.63	266	17	1.32	0.74 – 2.33
**Female sample**	2,518	201			3,266	181		
MHNW	404	18	Ref	Ref	1,361	67	Ref	Ref
MHOW	474	21	0.68	0.36 –1.28	729	52	1.43	0.99 – 2.05
MHO	486	33	0.87	0.48 – 1.56	193	9	1.12	0.55 – 2.24
MUNW	101	13	1.21	0.57 – 2.54	336	23	1.34	0.83 – 2.17
MUOW	279	35	1.32	0.74 – 2.35	381	17	0.90	0.53 – 1.54
MUO	774	81	1.01	0.60 – 1.72	266	13	1.08	0.59 – 1.95

b) Late-life	The HRS				The STR			
	N	Events	HR	95% CI	N	Events	HR	95% CI
**Total analytical sample**	8,837	2,077			7,212	1,669		
MHNW	1,152	284	Ref	Ref	2,014	475	Ref	Ref
MHOW	1,548	302	**0.84**	**0.72 – 0.99**	1,469	331	0.92	0.80 – 1.06
MHO	1,134	213	0.86	0.72 – 1.03	318	59	0.77	0.59 – 1.01
MUNW	727	238	1.07	0.89 – 1.27	1,034	264	1.12	0.97 – 1.31
MUOW	1,734	461	0.93	0.80 – 1.08	1,711	416	1.07	0.94 – 1.23
MUO	2,542	579	1.02	0.88 – 1.18	666	124	0.85	0.70 – 1.04
**Male sample**	3,759	847			3,349	710		
MHNW	392	101	Ref	Ref	837	183	Ref	Ref
MHOW	746	149	0.85	0.65 – 1.09	674	131	0.91	0.73 – 1.14
MHO	505	88	0.79	0.59 – 1.05	106	17	0.81	0.49 – 1.33
MUNW	230	70	1.07	0.78 – 1.47	500	121	1.22	0.97 – 1.54
MUOW	828	201	0.87	0.68 – 1.11	959	212	1.14	0.94 – 1.40
MUO	1,058	238	1.06	0.83 –1.35	273	46	1.02	0.73 – 1.41

**Female sample**	5,078	1,230			3,863	959		
MHNW	760	183	Ref	Ref	1,177	292	Ref	Ref
MHOW	802	153	0.83	0.66 – 1.03	795	200	0.94	0.78 – 1.13
MHO	629	125	0.94	0.74 – 1.18	212	42	0.76	0.55 – 1.05
MUNW	497	168	1.07	0.86 – 1.32	534	143	1.07	0.87 – 1.30
MUOW	906	260	1.00	0.83 – 1.22	752	204	1.03	0.86 – 1.24
MUO	1,484	341	0.99	0.82 – 1.19	393	78	0.79	0.61 – 1.01

Hazard rate ratios (HR) and 95% confidence intervals (CI) from Cox proportional hazard regression, showing risk of dementia in relation to MHOW, MHO, MUNW, MUOW, and MUO, compared to the reference category MHNW. Results are presented for measures taken in a) midlife (≤65 years) and b) late-life (>65 years), in the total analytical sample, the male analytical sample, and the female analytical sample. All models were adjusted for age, sex, smoking status, and education level. Models of the HRS were further adjusted for ethnicity, and models of the STR for sub-study. Bold numbers indicate statistical significance at the α=0.05 level.

Abbreviations: CI – Confidence interval, HR – Hazard ratio, HRS – Health and Retirement Study, N – Number of individuals, MHNW – Metabolically healthy normal weight, MHO – Metabolically healthy obesity, MHOW – Metabolically healthy overweight, MUNW – Metabolically unhealthy normal weight, MUO – Metabolically unhealthy obesity, MUOW – Metabolically unhealthy overweight, STR – Swedish Twin Registry.

Table S11. Risk of dementia in relation to obesity and metabolic health phenotypes, additionally adjusting for physical activity and alcohol consumption.

a) Midlife	The HRS				The STR			
	N	Events	HR	95% CI	N	Events	HR	95% CI
**Total analytical sample**	4,218	362			5,824	323		
MHNO	1,473	86	Ref	Ref	3,387	189	Ref	Ref
MHO	836	57	0.94	0.67 – 1.32	297	11	0.73	0.39 – 1.34
MUNO	723	90	1.28	0.94 – 1.73	1,613	93	1.05	0.81 – 1.35
MUO	1,186	129	1.02	0.77 – 1.36	527	30	1.26	0.85 – 1.87
**Male sample**	1,707	161			2,579	144		
MHNO	599	47	Ref	Ref	1,308	72	Ref	Ref
MHO	351	24	0.82	0.50 – 1.35	105	2	0.33	0.08 – 1.35
MUNO	345	42	1.04	0.68 – 1.60	902	53	1.08	0.76 – 1.56
MUO	412	48	0.87	0.57 – 1.33	264	17	1.62	0.94 – 2.79
**Female sample**	2,511	201			3,245	179		
MHNO	874	39	Ref	Ref	2,079	117	Ref	Ref
MHO	485	33	1.08	0.67 – 1.73	192	9	1.02	0.52 – 2.02
MUNO	378	48	**1.61**	**1.04 – 2.49**	711	40	1.02	0.71 – 1.47
MUO	774	81	1.24	0.83 – 1.86	263	13	1.02	0.57 – 1.83

b) Late-life	The HRS				The STR			
	N	Events	HR	95% CI	N	Events	HR	95% CI
**Total analytical sample**	8,797	2,067			6,717	1,547		
MHNO	2,686	582	Ref	Ref	3,151	725	Ref	Ref
MHO	1,128	211	0.92	0.78 – 1.08	285	54	0.81	0.61 – 1.07
MUNO	2,451	697	1.04	0.93 – 1.16	2,630	646	**1.12**	**1.01 – 1.25**
MUO	2,532	577	1.06	0.94 – 1.19	651	122	0.88	0.72 – 1.07
**Male sample**	3,738	842			3,165	667		
MHNO	1,130	249	Ref	Ref	1,397	289	Ref	Ref
MHO	501	87	0.86	0.67 – 1.10	96	15	0.80	0.48 – 1.35
MUNO	1,055	270	0.96	0.80 – 1.15	1,403	318	**1.22**	**1.04 – 1.43**
MUO	1,052	236	1.08	0.89 – 1.30	269	45	1.05	0.76 – 1.44
**Female sample**	5,059	1,225			3,552	880		
MHNO	1,556	333	Ref	Ref	1,754	436	Ref	Ref
MHO	627	124	0.99	0.81 – 1.23	189	39	0.79	0.57 – 1.10
MUNO	1,396	427	1.11	0.95 – 1.29	1,227	328	1.05	0.91 – 1.22
MUO	1,480	341	1.04	0.89 – 1.23	382	77	0.79	0.61 – 1.01

Hazard rate ratios (HR) and 95% confidence intervals (CI) from Cox proportional hazard regression, showing risk of dementia in relation to MUO, MUNO, and MHO compared to the reference category MHNO. Results are presented for measures taken in a) midlife (≤65 years) and b) late-life (>65 years), in the total analytical sample, the male analytical sample, and the female analytical sample. All models were adjusted for age, sex, smoking status, education level, physical activity, and alcohol consumption. Models of the HRS were further adjusted for ethnicity, and models of the STR for sub-study. Bold numbers indicate statistical significance at the α=0.05 level.

Abbreviations: CI – Confidence interval, HR – Hazard ratio, HRS – Health and Retirement Study, N – Number of individuals, MHNO – Metabolically healthy no obesity, MHO – Metabolically healthy obesity, MUNO – Metabolically unhealthy no obesity, MUO – Metabolically unhealthy obesity, STR – Swedish Twin Registry.

Table S12. Risk of dementia in relation to obesity and metabolic health phenotypes, additionally adjusting for depressive symptoms, physical activity, and alcohol consumption.

**a) Midlife**	**The HRS**				**The STR**			
	**N**	**Events**	**HR**	**95% CI**	**N**	**Events**	**HR**	**95% CI**
**Total analytical sample**	4,218	362			5,811	323		
MHNO	1,473	86	Ref	Ref	3,382	189	Ref	Ref
MHO	836	57	0.97	0.69 – 1.36	296	11	0.72	0.39 – 1.33
MUNO	723	90	1.31	0.97 – 1.77	1,607	93	1.04	0.80 – 1.34
MUO	1,186	129	1.05	0.79 – 1.39	526	30	1.26	0.85 – 1.86
**Male sample**	1,707	161			2,576	144		
MHNO	599	47	Ref	Ref	1,306	72	Ref	Ref
MHO	351	24	0.88	0.53 – 1.44	105	2	0.33	0.08 – 1.33
MUNO	345	42	1.07	0.70 – 1.64	902	53	1.07	0.75 – 1.54
MUO	412	48	0.90	0.59 – 1.36	263	17	1.63	0.95 – 2.81
**Female sample**	2,511	201			3,235	179		
MHNO	874	39	Ref	Ref	2,076	117	Ref	Ref
MHO	485	33	1.10	0.69 – 1.77	191	9	1.03	0.52 – 2.04
MUNO	378	48	**1.66**	**1.07 – 2.56**	705	40	1.01	0.70 – 1.46
MUO	774	81	1.26	0.84 – 1.88	263	13	1.00	0.56 – 1.80

**b) Late-life**	**The HRS**				**The STR**			
	**N**	**Events**	**HR**	**95% CI**	**N**	**Events**	**HR**	**95% CI**
**Total analytical sample**	8,797	2,067			6,678	1,537		
MHNO	2,686	582	Ref	Ref	3,138	722	Ref	Ref
MHO	1,128	211	0.88	0.75 – 1.04	283	53	0.79	0.60 – 1.05
MUNO	2,451	697	1.00	0.90 – 1.12	2,608	640	1.12	1.01 – 1.25
MUO	2,532	577	1.02	0.90 – 1.15	649	122	0.87	0.72 – 1.06
**Male sample**	3,738	842			3,152	665		
MHNO	1,130	249	Ref	Ref	1,395	288	Ref	Ref
MHO	501	87	0.85	0.66 – 1.09	96	15	0.81	0.48 –1.36
MUNO	1,055	270	0.94	0.79 – 1.13	1,392	317	**1.22**	**1.04 – 1.44**
MUO	1,052	236	1.06	0.88 – 1.28	269	45	1.05	0.76 – 1.44
**Female sample**	5,059	1,225			3,526	872		
MHNO	1,556	333	Ref	Ref	1,743	434	Ref	Ref
MHO	627	124	0.95	0.77 – 1.17	187	38	0.76	0.55 – 1.07
MUNO	1,396	427	1.06	0.91 – 1.23	1,216	323	1.04	0.90 – 1.21
MUO	1,480	341	1.00	0.85 – 1.18	380	77	0.78	0.61 – 1.00

Hazard rate ratios (HR) and 95% confidence intervals (CI) from Cox proportional hazard regression, showing risk of dementia in relation to MUO, MUNO, and MHO compared to the reference category MHNO. Results are presented for measures taken in a) midlife (≤65 years) and b) late-life (>65 years), in the total analytical sample, the male analytical sample, and the female analytical sample. All models were adjusted for age, sex, smoking status, education level, physical activity, alcohol consumption, and , depressive symptoms. Models of the HRS were further adjusted for ethnicity, and models of the STR for sub-study. Bold numbers indicate statistical significance at the α=0.05 level.

Abbreviations: CI – Confidence interval, HR – Hazard ratio, HRS – Health and Retirement Study, N – Number of individuals, MHNO – Metabolically healthy no obesity, MHO – Metabolically healthy obesity, MUNO – Metabolically unhealthy no obesity, MUO – Metabolically unhealthy obesity, STR – Swedish Twin Registry.

Table S13. Risk of dementia in relation to obesity and metabolic health phenotypes, additionally adjusting for depressive symptoms, physical activity, alcohol consumption, and Apolipoprotein E ε4.

a) Midlife	The HRS				The STR			
	N	Events	HR	95% CI	N	Events	HR	95% CI
**Total analytical sample**	3,877	335			4,992	283		
MHNO	1,375	80	Ref	Ref	2,851	159	Ref	Ref
MHO	776	53	0.97	0.68 –1.38	257	9	0.71	0.36 – 1.40
MUNO	645	81	1.27	0.93 –1.75	1,409	87	1.03	0.78 – 1.35
MUO	1,081	121	1.05	0.78 – 1.40	475	28	1.27	0.84 – 1.91
**Male sample**	1,562	146			2,241	136		
MHNO	561	45	Ref	Ref	1,109	65	Ref	Ref
MHO	319	21	0.82	0.48 – 1.39	92	2	0.35	0.08 – 1.43
MUNO	301	36	0.98	0.62 – 1.53	802	53	1.03	0.71 – 1.50
MUO	381	44	0.87	0.57 – 1.34	238	16	1.58	0.90 – 2.77
**Female sample**	2,315	189			2,751	147		
MHNO	814	35	Ref	Ref	1,742	94	Ref	Ref
MHO	457	32	1.17	0.72 – 1.92	165	7	1.09	0.50 – 2.36
MUNO	344	45	**1.72**	**1.09 – 2.72**	607	34	1.07	0.72 – 1.59
MUO	700	77	1.34	0.88 – 2.04	237	12	0.99	0.53 – 1.82

b) Late-life	The HRS				The STR			
	N	Events	HR	95% CI	N	Events	HR	95% CI
**Total analytical sample**	8,220	1,938			5,649	1,307		
MHNO	2,528	554	Ref	Ref	2,636	614	Ref	Ref
MHO	1,067	205	0.92	0.78 – 1.09	233	46	0.80	0.59 – 1.08
MUNO	2,266	644	0.96	0.86 – 1.08	2,211	540	1.08	0.96 – 1.21
MUO	2,359	535	0.99	0.87 – 1.12	569	107	0.85	0.69 – 1.05
**Male sample**	3,500	785			2,853	599		
MHNO	1,064	238	Ref	Ref	1,255	254	Ref	Ref
MHO	484	84	0.84	0.66 – 1.09	89	15	0.89	0.53 – 1.50
MUNO	973	242	0.87	0.72 – 1.05	1,262	287	**1.21**	**1.02 – 1.44**
MUO	979	221	1.01	0.83 – 1.22	247	43	1.10	0.80 – 1.53
**Female sample**	4,720	1,153			2,796	708		
MHNO	1,464	316	Ref	Ref	1,381	360	Ref	Ref
MHO	583	121	1.04	0.84 – 1.29	144	31	0.74	0.51 – 1.07
MUNO	1,293	402	1.04	0.89 – 1.21	949	253	0.97	0.82 – 1.14
MUO	1,380	314	0.98	0.83 – 1.17	322	64	**0.73**	**0.56 – 0.96**

Hazard rate ratios (HR) and 95% confidence intervals (CI) from Cox proportional hazard regression, showing risk of dementia in relation to MUO, MUNO, and MHO compared to the reference category MHNO. Results are presented for measures taken in a) midlife (≤65 years) and b) late-life (>65 years), in the total analytical sample, the male analytical sample, and the female analytical sample. All models were adjusted for age, sex, smoking status, education level, depression, physical activity, alcohol consumption, and Apolipoprotein E ε4. Models of the HRS were further adjusted for ethnicity, and models of the STR for sub-study. Bold numbers indicate statistical significance at the α=0.05 level.

Abbreviations: CI – Confidence interval, HR – Hazard ratio, HRS – Health and Retirement Study, N – Number of individuals, MHNO – Metabolically healthy no obesity, MHO – Metabolically healthy obesity, MUNO – Metabolically unhealthy no obesity, MUO – Metabolically unhealthy obesity, STR – Swedish Twin Registry.

Table S14. Risk of dementia in relation to obesity and metabolic health phenotypes, from competing risk regression modelling death as the competing event

a) Midlife	The HRS					The STR				
	**N**	**Events**	**Death**	**HR**	**95% CI**	**N**	**Death**	**Events**	**HR**	**95% CI**
**Total analytical sample**	4,233	362	265			5,855	327	534		
MHNO	1,480	86	62	Ref	Ref	3,404	192	250	Ref	Ref
MHO	839	57	39	0.96	0.68 – 1.35	298	11	28	0.68	0.37 – 1.26
MUNO	726	90	62	1.24	0.92 – 1.67	1,621	94	178	0.99	0.77 – 1.28
MUO	1,188	129	102	1.05	0.79 – 1.38	532	30	78	1.08	0.74 – 1.59

b) Late-life	The HRS					The STR				
	**N**	**Events**	**Death**	**HR**	**95% CI**	**N**	**Death**	**Events**	**HR**	**95% CI**
**Total analytical sample**	8,895	2,078	2,075			7,143	1,669	2,660		
MHNO	2,721	586	590	Ref	Ref	3,451	806	1,193	Ref	Ref
MHO	1,140	213	227	**0.78**	**0.68 – 0.90**	314	59	134	**0.70**	**0.53 – 0.92**
MUNO	2,477	700	640	1.01	0.92 – 1.12	2,719	680	1,050	1.06	0.95 – 1.18
MUO	2,557	579	618	**0.78**	**0.71 – 0.87**	659	124	283	**0.72**	**0.59 – 0.88**

Hazard rate ratios (HR) and 95% confidence intervals (CI) from Fine-Gray subdistribution hazards regression, showing risk of dementia in relation to MUO, MUNO, and MHO compared to the reference category MHNO. Results are presented for measures taken in a) midlife (≤65 years) and b) late-life (>65 years) in the total analytical sample. All models were adjusted for age, sex, smoking status, education level, depression, physical activity, alcohol consumption, and Apolipoprotein E ε4. Models of the HRS were further adjusted for ethnicity, and models of the STR for sub-study. Bold numbers indicate statistical significance at the α=0.05 level.

Abbreviations: CI – Confidence interval, HR – Hazard ratio, HRS – Health and Retirement Study, N – Number of individuals, MHNO – Metabolically healthy no obesity, MHO – Metabolically healthy obesity, MUNO – Metabolically unhealthy no obesity, MUO – Metabolically unhealthy obesity, STR – Swedish Twin Registry.
