# Supplementary material for: Dementia risk by metabolic health and obesity in two prospective cohorts
Source: BMC Med. 2026 Jun 19;24:366. doi: 10.1186/s12916-026-05002-8 (PMC13292518; doi:10.1186/s12916-026-05002-8)
Supplement: Supplementary file 2 — Supplementary Material 2 [file 12916_2026_5002_MOESM2_ESM.docx]

**Additional file 1**

**Dementia Risk by Metabolic Health and Obesity in Two Prospective Cohorts**

Martin Nakash, Elsa Ojalehto Lindfors, Yiqiang Zhan, Anna K. Dahl Aslan, Chandra A. Reynolds, Peggy Ler, Ida K. Karlsson

**Contents**

[Table S1. ICD-codes used to identify dementia in the Swedish Twin Registry. 1](#_Toc1822842440)

[Table S2. ATC-codes for identification of dementia medication in the Swedish Twin Registry. 2](#_Toc1961551042)

[Table S3. Diagnostic criteria for the metabolic health variables. 3](#_Toc1642865019)

[Table S4. Creation of physical activity measure in the Swedish Twin Registry studies. 3](#_Toc1668585298)

[Table S5. Risk of dementia in relation to obesity and metabolic health phenotypes. 4](#_Toc1849666953)

[Table S6. Separate, joint, and interaction models of risk of dementia in relation to obesity and poor metabolic health 5](#_Toc860685042)

[Table S7. Risk of dementia in relation to obesity and metabolic health phenotypes measured at ages <63 years for midlife and ages >67 for late life. 7](#_Toc2090447113)

[Table S8. Risk of dementia in relation to obesity and metabolic health, where metabolically unhealthy status was defined as having at least one unhealthy metabolic component. 8](#_Toc1325941634)

[Table S9. Risk of dementia in relation to obesity and metabolic health in the STR, where metabolically unhealthy status was defined without triglycerides (based on hypertension, hyperglycemia, and dyslipidemia (HDL-C)) 9](#_Toc1441004337)

[Table S10. Risk of dementia in relation to obesity and metabolic health phenotypes, classified as six categories. 11](#_Toc1573679405)

[Table S11. Risk of dementia in relation to obesity and metabolic health phenotypes, additionally adjusting for physical activity and alcohol consumption. 12](#_Toc2135214768)

[Table S12. Risk of dementia in relation to obesity and metabolic health phenotypes, additionally adjusting for depressive symptoms, physical activity, and alcohol consumption. 13](#_Toc817007222)

[Table S13. Risk of dementia in relation to obesity and metabolic health phenotypes, additionally adjusting for depressive symptoms, physical activity, alcohol consumption, and Apolipoprotein E ε4. 15](#_Toc1985728603)

[Table S14. Risk of dementia in relation to obesity and metabolic health phenotypes, from competing risk regression modelling death as the competing event 16](#_Toc1960719545)

# Table S1. ICD-codes used to identify dementia in the Swedish Twin Registry.

| **ICD-7** | **ICD-8** | **ICD-9*** | **ICD-10** |
| --- | --- | --- | --- |
| **(used before 1969)** | **(used 1969-1986)** | **(used 1987-1996)** | **(used 1997 and onwards)** |
| **304** Senile psychosis | **290** Senile and presenile dementia | **290** Senile and presenile organic psychotic condition | **F00** Dementia in Alzheimer's disease |
| **305** Presenile psychosis | **293.0** Cerebral arteriosclerosis | **294B/ 294.1** Dementia in conditions classified elsewhere | **F01** Vascular dementia |
| **306** Psychosis with cerebral arteriosclerosis | **293.1** Other cerebrovascular disturbances | **331A/ 331.0**  Alzheimer's disease | **F02** Dementia in other diseases classified elsewhere |
|  |  | **331B/ 331.1** Pick's disease | **F03** Unspecified dementia |
|  |  | **331C/ 331.2** Senile degeneration of brain | **F051** Delirium superimposed on dementia |
|  |  | **331X/ 331.9** Cerebral degeneration, unspecified | **G30** Alzheimer's disease |
|  |  |  | **G311** Senile degeneration of brain, not elsewhere classified |
|  |  |  | **G318A** Other specified degenerative diseases of nervous system: Lewy body dementia |

ICD-codes used to identify dementia diagnosis. By using the unique personal identification number assigned to all Swedish residents, diagnoses were obtained from the National Patient Register (NPR) and the Cause of Death Register (CDR) [18].

* In the Swedish adaptation of ICD-9, the 4^th^ digit was replaced with a letter. While the Swedish National Patient Register used the Swedish adaptation of ICD-9, the ICD versions was used in Cause of Death Registries, therefore, both versions of the codes are reported here.

# Table S2. ATC-codes for identification of dementia medication in the Swedish Twin Registry.

| **N06DA** Anticholinesterases |
| --- |
| N06DA02 Donepezil N06DA03 Rivastigmine N06DA04 Galantamine  (N06DA01 Tacrine and N06DA05 Ipidacrine not prescribed in Sweden) |
| **N06DX** Other anti-dementia drugs |
| N06DX01 Memantine  (N06DX02 Ginkgo folium not prescribed in Sweden) |

The ATC-codes for dementia medication. Prescribed dementia medication from the Prescribed Drug Register was used as a proxy to the diagnoses [17].

# Table S3. Diagnostic criteria for the metabolic health variables.

| **Metabolic health variables** | **Diagnostic criteria** | |
| --- | --- | --- |
|  | **The HRS** | **The STR** |
| **Hypertension** | SBP ≥130 mmHg or | SBP ≥130 mmHg or |
|  | DBP ≥85 mmHg | DBP ≥85 mmHg |
|  | Self-reported hypertension | Self-reported hypertension |
| **Hyperglycemia** | HbA1c >5.7% or | HbA1c >5.7% or |
|  | Self-reported diabetes | Self-reported diabetes or |
|  |  | Fasting BG ≥6.1 mmol/L or |
|  |  | Non-fasting BG ≥7.0 mmol/L |
| **Dyslipidemia (TG)** | No data | Fasting TG ≥1.7 mmol/ or |
|  |  | Non-fasting TG ≥2.1 mmol/L |
|  |  | Use of cholesterol lowering  medication |
| **Dyslipidemia (HDL-C)** | HDL-C <40 mg/dL in males or | HDL-C <1.03 mmol/L in males or |
|  | HDL-C <50 mg/dL in females | HDL-C <1.30 in females |
|  |  | Use of cholesterol lowering  medication. |
| **Metabolically unhealthy status** | ≥2 of 3 metabolic health variables | ≥2 of 4 metabolic health variables |

The diagnostic criteria for hypertension, hyperglycemia, and dyslipidemia used in the HRS and the STR.

Abbreviations. BG – Blood glucose. DBP – Diastolic blood pressure. HbA1c – Hemoglobin A1c. HDL-C – High-Density Lipoprotein Cholesterol. HRS – Health and Retirement Study. SBP – Systolic blood pressure. STR – Swedish Twin Registry. TG – Triglycerides.

# Table S4. Creation of physical activity measure in the Swedish Twin Registry studies.

| **Physical activity** | **SALT: How much do you exercise?** | **SALT:**  **Of these 7 alternatives, which fits your annual exercise pattern?** | **SATSA: Please state below how much exercise you get during your leisure time.** | **GENDER: Leisure activities - do you exercise** | **GENDER: Leisure activities - do you walk** |
| --- | --- | --- | --- | --- | --- |
| **0** | 0: Almost no exercise | 0: Almost never exercise | 1: I hardly get any exercise at all | 5: Never | 5: Never |
|  |  | 1: Much less exercise than average | 2: I get very little exercise |  | 4: more seldom than once/week |
|  |  | 2: Less exercise than average |  |  | 3: once or a few/month |
| **1** | 1: Light exercise, e.g., regular promenades or light garden walk | 3: Average amount of exercise | 3: I get little exercise | 4: more seldom than once/week | 2: once or a few/week |
|  |  |  | 4: I don’t get very much exercise | 3: once or a few/month | 1: daily |
| **2** | 2: Regular medium exercise | 4: More exercise than average | 5: I get quite a lot of exercise | 2: once or a few/week |  |
|  | 3: Hard physical exercise | 5: Much more exercise than average | 6: I get a lot of exercise | 1: daily |  |
|  |  | 6: Maximum amount of exercise | 7: I get very much exercise |  |  |

A new three-level variable for physical activity was created, based on different questions from the sub-studies from the Swedish Twin Registry. In SALT, participants born 1925 or earlier were asked how much they exercise (first column), and participants born after 1925 were asked about their annual exercise pattern (second column). In SATSA, the first, second, third, seventh, eighth, and ninth questionnaire asked about leisure time exercise (third column). The response closest in age to the biomarker measure was used. In GENDER, the first questionnaire included questions on leisure time exercise and walking.

# Table S5. Risk of dementia in relation to obesity and metabolic health phenotypes.

| 1. **Midlife** | **The HRS** | | | | **The STR** | | | |
| --- | --- | --- | --- | --- | --- | --- | --- | --- |
|  | **N** | **Events** | **HR** | **95% CI** | **N** | **Events** | **HR** | **95% CI** |
| **Total analytical sample** | 4,233 | 362 |  | | 5,856 | 327 |  | |
| MHNO | 1,480 | 86 | Ref | Ref | 3,404 | 192 | Ref | Ref |
| MHO | 839 | 57 | 0.96 | 0.68 – 1.35 | 298 | 11 | 0.70 | 0.38 – 1.30 |
| MUNO | 726 | 90 | 1.33 | 0.98 – 1.79 | 1,622 | 94 | 1.03 | 0.80 – 1.32 |
| MUO | 1,188 | 129 | 1.08 | 0.81 – 1.43 | 532 | 30 | 1.19 | 0.81 – 1.75 |
| **Male sample** | 1,715 | 161 |  | | 2,590 | 146 |  | |
| MHNO | 602 | 47 | Ref | Ref | 1,314 | 73 | Ref | Ref |
| MHO | 353 | 24 | 0.85 | 0.52 – 1.40 | 105 | 2 | 0.32 | 0.08 – 1.31 |
| MUNO | 346 | 42 | 1.12 | 0.73 – 1.71 | 905 | 54 | 1.08 | 0.76 – 1.55 |
| MUO | 414 | 48 | 0.93 | 0.61 – 1.41 | 266 | 17 | 1.52 | 0.88 – 2.60 |
| **Female sample** | 2,518 | 201 |  | | 3,266 | 181 |  | |
| MHNO | 878 | 39 | Ref | Ref | 2,090 | 119 | Ref | Ref |
| MHO | 486 | 33 | 1.09 | 0.68 – 1.75 | 193 | 9 | 0.97 | 0.49 – 1.91 |
| MUNO | 380 | 48 | **1.62** | **1.05 – 2.49** | 717 | 40 | 0.97 | 0.67 – 1.39 |
| MUO | 774 | 81 | 1.27 | 0.86 – 1.89 | 266 | 13 | 0.93 | - 1. – 1.66 |

| 1. **Late-life** | **The HRS** | | | | **The STR** | | | |
| --- | --- | --- | --- | --- | --- | --- | --- | --- |
|  | **N** | **Events** | **HR** | **95% CI** | **N** | **Events** | **HR** | **95% CI** |
| **Total analytical sample** | 8,841 | 2,078 |  | | 7,212 | 1,669 |  | |
| MHNO | 2,702 | 586 | Ref | Ref | 3,483 | 806 | Ref | Ref |
| MHO | 1,134 | 213 | 0.94 | 0.81 – 1.11 | 318 | 59 | 0.80 | 0.61 – 1.04 |
| MUNO | 2,463 | 700 | 1.07 | 0.95 – 1.19 | 2,745 | 680 | **1.13** | **1.02 – 1.25** |
| MUO | 2,542 | 579 | 1.12 | 0.99 – 1.26 | 666 | 124 | 0.88 | 0.73 – 1.07 |
| **Male sample** | 3,759 | 847 |  | | 3,349 | 710 |  | |
| MHNO | 1,138 | 250 | Ref | Ref | 1,511 | 314 | Ref | Ref |
| MHO | 505 | 88 | 0.88 | 0.68 – 1.12 | 106 | 17 | 0.84 | 0.52 – 1.37 |
| MUNO | 1,058 | 271 | 1.01 | 0.84 – 1.21 | 1,459 | 333 | **1.22** | **1.04 – 1.42** |
| MUO | 1,058 | 238 | 1.18 | 0.98 – 1.42 | 273 | 46 | 1.06 | 0.77 – 1.45 |
| **Female sample** | 5,082 | 1,231 |  | | 3,863 | 959 |  | |
| MHNO | 1,564 | 336 | Ref | Ref | 1,972 | 492 | Ref | Ref |
| MHO | 629 | 125 | 1.03 | 0.84 – 1.27 | 212 | 42 | 0.78 | 0.57 – 1.07 |
| MUNO | 1,405 | 429 | 1.13 | 0.98 – 1.31 | 1,286 | 347 | 1.07 | 0.93 – 1.23 |
| MUO | 1,484 | 341 | 1.09 | 0.93 – 1.28 | 393 | 78 | 0.81 | 0.63 – 1.03 |

Hazard rate ratios (HR) and 95% confidence intervals (CI) from Cox proportional hazard regression, showing risk of dementia in relation to MUO, MUNO, and MHO compared to the reference category MHNO. Results are presented for measures taken in a) midlife (age ≤65 years) late-life (>65 years), in the total analytical sample, the male analytical sample, and the female analytical sample. All models were adjusted for age, sex, smoking status, and education level. Models of the HRS were further adjusted for ethnicity, and models of the STR for sub-study. Bold numbers indicate statistical significance at the α=0.05 level.

Abbreviations: CI – Confidence interval, HR – Hazard ratio, HRS – Health and Retirement Study, N – Number of individuals, MHNO – Metabolically healthy no obesity, MHO – Metabolically healthy obesity, MUNO – Metabolically unhealthy no obesity, MUO – Metabolically unhealthy obesity, STR – Swedish Twin Registry**.**

# Table S6. Separate, joint, and interaction models of risk of dementia in relation to obesity and poor metabolic health

| 1. **Midlife** |  | **The HRS** | | **The STR** | |
| --- | --- | --- | --- | --- | --- |
| **Total analytical sample** |  | **HR** | **95% CI** | **HR** | **95% CI** |
| Model 1: Obesity | Obesity | 0.91 | 0.73 – 1.12 | 0.99 | 0.72 – 1.38 |
| Model 2: MU | MU | 1.19 | 0.96 – 1.48 | 1.09 | 0.87 – 1.37 |
| Model 3: Joint model, obesity and MU | Obesity | 0.87 | 0.70 – 1.08 | 0.97 | 0.69 – 1.36 |
|  | MU | 1.23 | 0.98 – 1.53 | 1.09 | 0.86 – 1.38 |
| Model 4: Interaction model, obesity and MU | Obesity | 0.96 | 0.68 – 1.35 | 0.70 | 0.38 – 1.30 |
|  | MU | 1.33 | 0.98 – 1.79 | 1.03 | 0.80 – 1.32 |
|  | Obesity*MU | 0.85 | 0.55 – 1.31 | 1.64 | 0.79 – 3.43 |
| **Male sample** |  |  |  |  |  |
| Model 1: Obesity | Obesity | 0.86 | 0.62 – 1.18 | 1.05 | 0.64 – 1.72 |
| Model 2: MU | MU | 1.07 | 0.78 – 1.48 | 1.23 | 0.88 – 1.71 |
| Model 3: Joint model, obesity and MU | Obesity | 0.84 | 0.61 – 1.16 | 1.01 | 0.61 – 1.65 |
|  | MU | 1.11 | 0.80 – 1.54 | 1.23 | 0.88 – 1.72 |
| Model 4: Interaction model, obesity and MU | Obesity | 0.85 | 0.52 – 1.40 | 0.32 | 0.08 – 1.31 |
|  | MU | 1.12 | 0.73 – 1.71 | 1.08 | 0.76 – 1.55 |
|  | Obesity*MU | 0.97 | 0.51 – 1.87 | 4.36 | 0.96 – 19.7 |
| **Female sample** |  |  |  |  |  |
| Model 1: Obesity | Obesity | 0.95 | 0.71 – 1.27 | 0.96 | 0.61 – 1.50 |
| Model 2: MU | MU | 1.33 | 0.99 – 1.80 | 0.96 | 0.69 – 1.33 |
| Model 3: Joint model, obesity and MU | Obesity | 0.89 | 0.66 – 1.19 | 0.97 | 0.61 – 1.53 |
|  | MU | **1.37** | **1.01 – 1.86** | 0.97 | 0.69 – 1.35 |
| Model 4: Interaction model, obesity and MU | Obesity | 1.09 | 0.68 – 1.75 | 0.97 | 0.49 – 1.91 |
|  | MU | **1.62** | **1.05 – 2.49** | 0.97 | 0.67 – 1.39 |
|  | Obesity*MU | 0.72 | 0.40 – 1.30 | 1.00 | 0.40 – 2.52 |

| 1. **Late-life** |  | **The HRS** | | **The STR** | |
| --- | --- | --- | --- | --- | --- |
| **Total analytical sample** |  | **HR** | **95% CI** | **HR** | **95% CI** |
| Model 1: Obesity | Obesity | 1.02 | 0.94 – 1.12 | **0.81** | **0.69 – 0.94** |
| Model 2: MU | MU | **1.11** | **1.01 – 1.21** | **1.10** | **1.00 – 1.22** |
| Model 3: Joint model, obesity and MU | Obesity | 1.01 | 0.92 – 1.11 | **0.79** | **0.67 – 0.92** |
|  | MU | **1.10** | **1.01 – 1.21** | **1.13** | **1.02 – 1.25** |
| Model 4: Interaction model, obesity and MU | Obesity | 0.94 | 0.81 – 1.11 | 0.80 | 0.61 – 1.04 |
|  | MU | 1.07 | 0.95 – 1.19 | **1.13** | **1.02 – 1.26** |
|  | Obesity*MU | 1.11 | 0.91 – 1.34 | 0.98 | 0.71 – 1.36 |
| **Male sample** |  |  |  |  |  |
| Model 1: Obesity | Obesity | 1.07 | 0.93 – 1.23 | 0.89 | 0.69 – 1.16 |
| Model 2: MU | MU | 1.12 | 0.97 – 1.30 | **1.21** | **1.04 – 1.40** |
| Model 3: Joint model, obesity and MU | Obesity | 1.05 | 0.91 – 1.22 | 0.86 | 0.66 – 1.12 |
|  | MU | 1.11 | 0.96 – 1.29 | **1.22** | **1.05 – 1.42** |
| Model 4: Interaction model, obesity and MU | Obesity | 0.88 | 0.68 – 1.12 | 0.84 | 0.52 – 1.37 |
|  | MU | 1.01 | 0.84 – 1.21 | **1.22** | **1.04 – 1.43** |
|  | Obesity*MU | 1.33 | 0.98 – 1.80 | 1.03 | 0.58 – 1.84 |
| **Female sample** |  |  |  |  |  |
| Model 1: Obesity | Obesity | 1.00 | 0.89 – 1.13 | **0.77** | **0.64 – 0.94** |
| Model 2: MU | MU | 1.10 | 0.98 – 1.24 | 1.04 | 0.91 – 1.18 |
| Model 3: Joint model, obesity and MU | Obesity | 0.99 | 0.87 – 1.12 | **0.76** | **0.63 – 0.92** |
|  | MU | 1.11 | 0.98 – 1.25 | 1.07 | 0.94 – 1.22 |
| Model 4: Interaction model, obesity and MU | Obesity | 1.03 | 0.84 – 1.27 | 0.78 | 0.57 – 1.07 |
|  | MU | 1.13 | 0.98 – 1.31 | 1.07 | 0.93 – 1.23 |
|  | Obesity*MU | 0.94 | 0.73 – 1.21 | 0.97 | 0.65 – 1.45 |

Hazard rate ratios (HR) and 95% confidence intervals (CI) from Cox proportional hazard regression modelling 1) obesity separately, 2) metabolically unhealthy status (MU) separately, 3) obesity and MU jointly, and 4) an interaction between obesity and MU. Results are presented for measures taken in a) midlife (≤65 years) and b) late-life (>65 years), in the HRS and the STR. All models were adjusted for age, sex, smoking status, and education level. Models of the HRS were further adjusted for ethnicity, and models of the STR for sub-study. Bold numbers indicate statistical significance at the α=0.05 level.

Abbreviations: CI – Confidence interval, HR – Hazard ratio, HRS – Health and Retirement Study, MU – Metabolically unhealthy, STR – Swedish Twin Registry**.**

# Table S7. Risk of dementia in relation to obesity and metabolic health phenotypes measured at ages <63 years for midlife and ages >67 for late life.

| 1. **Midlife** | **The HRS** | | | | **The STR** | | | |
| --- | --- | --- | --- | --- | --- | --- | --- | --- |
|  | **N** | **Events** | **HR** | **95% CI** | **N** | **Events** | **HR** | **95% CI** |
| **Total analytical sample** | 3,356 | 248 |  | | 4,545 | 222 |  | |
| MHNO | 1,208 | 59 | Ref | Ref | 2,699 | 127 | Ref | Ref |
| MHO | 696 | 42 | 1.12 | 0.75 – 1.68 | 230 | 9 | 0.89 | 0.45 – 1.76 |
| MUNO | 543 | 57 | 1.20 | 0.83 – 1.74 | 1,202 | 64 | 1.27 | 0.93 – 1.74 |
| MUO | 909 | 90 | 1.31 | 0.93 – 1.84 | 414 | 22 | 1.29 | 0.82 – 2.04 |
| **Male sample** | 1,372 | 114 |  | | 2,002 | 92 |  | |
| MHNO | 483 | 31 | Ref | Ref | 1,028 | 38 | Ref | Ref |
| MHO | 300 | 19 | 1.08 | 0.61 – 1.94 | 76 | 2 | 0.68 | 0.16 – 2.84 |
| MUNO | 268 | 29 | 1.00 | 0.59 – 1.94 | 690 | 40 | **1.80** | **1.14 – 2.84** |
| MUO | 321 | 35 | 1.16 | 0.71 – 1.91 | 208 | 12 | **2.04** | **1.04 – 3.99** |
| **Female sample** | 1,984 | 134 |  | | 2,543 | 130 |  | |
| MHNO | 725 | 28 | Ref | Ref | 1,671 | 89 | Ref | Ref |
| MHO | 396 | 23 | 1.23 | 0.70 – 2.16 | 154 | 7 | 1.01 | 0.47 – 2.19 |
| MUNO | 275 | 28 | 1.48 | 0.86 – 2.55 | 512 | 24 | 0.95 | 0.60 – 1.50 |
| MUO | 588 | 55 | 1.54 | 0.96 – 2.48 | 206 | 10 | 0.95 | 0.49 – 1.82 |

| 1. **Late-life** | **The HRS** | | | | **The STR** | | | |
| --- | --- | --- | --- | --- | --- | --- | --- | --- |
|  | **N** | **Events** | **HR** | **95% CI** | **N** | **Events** | **HR** | **95% CI** |
| **Total analytical sample** | 6,467 | 1,827 |  | | 5,257 | 1,405 |  | |
| MHNO | 2,034 | 532 | Ref | Ref | 2,499 | 686 | Ref | Ref |
| MHO | 796 | 187 | 0.94 | 0.80 – 1.11 | 242 | 50 | 0.76 | 0.57 – 1.01 |
| MUNO | 1,890 | 624 | 1.04 | 0.93 –1.17 | 2,044 | 570 | 1.09 | 0.97 – 1.22 |
| MUO | 1,747 | 484 | 1.08 | 0.96 – 1.23 | 472 | 99 | 0.83 | 0.67 – 1.03 |
| **Male sample** | 2,793 | 746 |  | | 2,460 | 597 |  | |
| MHNO | 874 | 225 | Ref | Ref | 1,105 | 272 | Ref | Ref |
| MHO | 373 | 80 | 0.85 | 0.66 – 1.10 | 78 | 14 | 0.77 | 0.45 – 1.32 |
| MUNO | 803 | 240 | 0.97 | 0.80 – 1.16 | 1,086 | 277 | 1.15 | 0.97 – 1.37 |
| MUO | 743 | 201 | 1.13 | 0.93 – 1.37 | 191 | 34 | 0.93 | 0.65 – 1.33 |
| **Female sample** | 3,674 | 1,081 |  | | 2,797 | 808 |  | |
| MHNO | 1,160 | 307 | Ref | Ref | 1,394 | 414 | Ref | Ref |
| MHO | 423 | 107 | 1.05 | 0.84 – 1.30 | 164 | 36 | 0.75 | 0.53 – 1.06 |
| MUNO | 1,087 | 384 | 1.10 | 0.95 – 1.28 | 958 | 293 | 1.05 | 0.90 – 1.22 |
| MUO | 1,004 | 283 | 1.06 | 0.90 – 1.26 | 281 | 65 | 0.79 | 0.61 – 1.03 |

Hazard rate ratios (HR) and 95% confidence intervals (CI) from Cox proportional hazard regression, showing risk of dementia in relation to MUO, MUNO, and MHO compared to the reference category MHNO. Results are presented for measures taken at a) ages <63 for midlife, and b) ages >67 years for late life, in the total analytical sample, the male analytical sample, and the female analytical sample. All models were adjusted for age, sex, smoking status, and education level. Models of the HRS were further adjusted for ethnicity, and models of the STR for sub-study. Bold numbers indicate statistical significance at the α=0.05 level.

Abbreviations: CI – Confidence interval, HR – Hazard ratio, HRS – Health and Retirement Study, N – Number of individuals, MHNO – Metabolically healthy no obesity, MHO – Metabolically healthy obesity, MUNO – Metabolically unhealthy no obesity, MUO – Metabolically unhealthy obesity, STR – Swedish Twin Registry**.**

# Table S8. Risk of dementia in relation to obesity and metabolic health, where metabolically unhealthy status was defined as having at least one unhealthy metabolic component.

| 1. **Midlife** | **The HRS** | | | | **The STR** | | | |
| --- | --- | --- | --- | --- | --- | --- | --- | --- |
|  | **N** | **Events** | **HR** | **95% CI** | **N** | **Events** | **HR** | **95% CI** |
| **Total analytical sample** | 4,233 | 362 |  | | 5,856 | 327 |  | |
| MHNO | 547 | 13 | Ref | Ref | 1,245 | 56 | Ref | Ref |
| MHO | 168 | 8 | 1.76 | 0.73 –4.25 | 51 | 3 | 1.57 | 0.49 – 5.03 |
| MUNO | 1,659 | 163 | **2.35** | **1.33 – 4.15** | 3,781 | 230 | 0.94 | 0.70 –1.26 |
| MUO | 1,859 | 178 | **1.96** | **1.11 – 3.47** | 779 | 38 | 0.92 | 0.60 – 1.38 |
| **Male sample** | 1,715 | 161 |  | | 2,590 | 146 |  | |
| MHNO | 189 | 7 | Ref | Ref | 436 | 25 | Ref | Ref |
| MHO | 59 | 3 | 1.22 | 0.32 – 4.76 | 13 | 1 | 1.06 | 0.14 – 7.90 |
| MUNO | 759 | 82 | 1.88 | 0.86 – 4.11 | 1,783 | 102 | 0.76 | 0.49 – 1.18 |
| MUO | 708 | 69 | 1.53 | 0.69 – 3.37 | 358 | 18 | 0.83 | 0.45 – 1.53 |
| **Female sample** | 2,518 | 201 |  | | 3,266 | 181 |  | |
| MHNO | 358 | 6 | Ref | Ref | 809 | 31 | Ref | Ref |
| MHO | 109 | 5 | 2.33 | 0.71 – 7.68 | 38 | 2 | 1.95 | 0.47 – 8.17 |
| MUNO | 900 | 81 | **2.95** | **1.28 – 6.82** | 1,998 | 128 | 1.10 | 0.74 – 1.63 |
| MUO | 1,151 | 109 | **2.51** | **1.09 – 5.80** | 421 | 20 | 0.98 | 0.56 – 1.73 |

| 1. **Late-life** | **The HRS** | | | | **The STR** | | | |
| --- | --- | --- | --- | --- | --- | --- | --- | --- |
|  | **N** | **Events** | **HR** | **95% CI** | **N** | **Events** | **HR** | **95% CI** |
| **Total analytical sample** | 8,841 | 2,078 |  | | 7,212 | 1,669 |  | |
| MHNO | 655 | 118 | Ref | Ref | 684 | 155 | Ref | Ref |
| MHO | 156 | 28 | 0.99 | 0.65 – 1.50 | 28 | 10 | 1.84 | 0.97 – 3.50 |
| MUNO | 4,510 | 1,168 | 0.98 | 0.81 – 1.19 | 5,544 | 1,331 | 0.98 | 0.83 –1.16 |
| MUO | 3,520 | 764 | 1.01 | 0.83 – 1.23 | 956 | 173 | **0.77** | **0.62 – 0.96** |
| **Male sample** | 3,759 | 847 |  | | 3,349 | 710 |  | |
| MHNO | 248 | 50 | Ref | Ref | 302 | 68 | Ref | Ref |
| MHO | 65 | 13 | 0.95 | 0.51 – 1.76 | 7 | 2 | 2.11 | 0.52 – 8.63 |
| MUNO | 1,948 | 471 | 0.91 | 0.68 – 1.23 | 2,668 | 579 | 0.99 | 0.77 – 1.27 |
| MUO | 1,498 | 313 | 0.99 | 0.73 – 1.34 | 372 | 61 | 0.87 | 0.61 – 1.23 |
| **Female sample** | 5,082 | 1,231 |  | | 3,863 | 959 |  | |
| MHNO | 407 | 68 | Ref | Ref | 382 | 87 | Ref | Ref |
| MHO | 91 | 15 | 1.01 | 0.57 – 1.77 | 21 | 8 | 1.71 | 0.83 – 3.53 |
| MUNO | 2,562 | 697 | 1.05 | 0.81 – 1.35 | 2,876 | 752 | 0.98 | 0.78 – 1.22 |
| MUO | 2,022 | 451 | 1.05 | 0.81 – 1.36 | 584 | 112 | **0.73** | **0.55 – 0.97** |

Hazard rate ratios (HR) and 95% confidence intervals (CI) from Cox proportional hazard regression, showing risk of dementia in relation to MUO, MUNO, and MHO compared to the reference category MHNO. Metabolically healthy status was defined as having no hypertension, hyperglycemia or dyslipidemia. Results are presented for measures taken in a) midlife (≤65 years) and b) late-life (>65 years), in the total analytical sample, the male analytical sample, and the female analytical sample. All models were adjusted for age, sex, smoking status, and education level. Models of the HRS were further adjusted for ethnicity, and models of the STR for sub-study. Bold numbers indicate statistical significance at the α=0.05 level.

Abbreviations: CI – Confidence interval, HR – Hazard ratio, HRS – Health and Retirement Study, N – Number of individuals, MHNO – Metabolically healthy no obesity, MHO – Metabolically healthy obesity, MUNO – Metabolically unhealthy no obesity, MUO – Metabolically unhealthy obesity, STR – Swedish Twin Registry**.**

# Table S9. Risk of dementia in relation to obesity and metabolic health in the STR, where metabolically unhealthy status was defined without triglycerides (based on hypertension, hyperglycemia, and dyslipidemia (HDL-C))

|  | 1. **Midlife** | | | | 1. **Late-life** | | | |
| --- | --- | --- | --- | --- | --- | --- | --- | --- |
|  | **N** | **Events** | **HR** | **95% CI** | **N** | **Events** | **HR** | **95% CI** |
| **Total analytical sample** | 5,856 | 327 |  |  | 7,228 | 1,671 |  |  |
| MHNO | 3,872 | 219 | Ref | Ref | 4,252 | 983 | Ref | Ref |
| MHO | 417 | 21 | 0.99 | 0.63 – 1.55 | 468 | 91 | 0.83 | 0.67 – 1.03 |
| MUNO | 1,154 | 67 | 1.02 | 0.77 – 1.34 | 1,989 | 505 | **1.22** | **1.10 – 1.37** |
| MUO | 413 | 20 | 1.01 | 0.64 – 1.60 | 519 | 92 | 0.89 | 0.72 – 1.10 |
| **Male sample** | 2,590 | 146 |  |  | 3,356 | 710 |  |  |
| MHNO | 1,594 | 89 | Ref | Ref | 1,881 | 391 | Ref | Ref |
| MHO | 155 | 6 | 0.73 | 0.32 – 1.68 | 171 | 33 | 1.02 | 0.71 – 1.47 |
| MUNO | 625 | 38 | 1.10 | 0.75 – 1.62 | 1,093 | 256 | **1.29** | **1.10 – 1.51** |
| MUO | 216 | 13 | 1.39 | 0.77 – 2.52 | 211 | 30 | 0.94 | 0.64 – 1.36 |
| **Female sample** | 3,266 | 181 |  |  | 3,872 | 961 |  |  |
| MHNO | 2,278 | 130 | Ref | Ref | 2,371 | 592 | Ref | Ref |
| MHO | 262 | 15 | 1.16 | 0.68 – 1.98 | 297 | 58 | **0.75** | **0.57 – 0.98** |
| MUNO | 529 | 29 | 0.94 | 0.62 – 1.40 | 896 | 249 | **1.18** | **1.01 – 1.37** |
| MUO | 197 | 7 | 0.68 | 0.31 – 1.45 | 308 | 62 | 0.88 | 0.67 –1.14 |

Hazard rate ratios (HR) and 95% confidence intervals (CI) from Cox proportional hazard regression, showing risk of dementia in relation to MUO, MUNO, and MHO compared to the reference category MHNO. Metabolically healthy status was defined as having maximum one metabolic component out of hypertension, hyperglycemia, or dyslipidemia based on HDL-C (excluding triglycerides). Results are presented for measures taken in a) midlife (≤65 years) and b) late-life (>65 years), in the total analytical sample, the male analytical sample, and the female analytical sample. All models were adjusted for age, sex, smoking status, education level, and sub-study. Bold numbers indicate statistical significance at the α=0.05 level.

Abbreviations: CI – Confidence interval, HR – Hazard ratio, N – Number of individuals, MHNO – Metabolically healthy no obesity, MHO – Metabolically healthy obesity, MUNO – Metabolically unhealthy no obesity, MUO – Metabolically unhealthy obesity, TG – Triglycerides**.**

# Table S10. Risk of dementia in relation to obesity and metabolic health phenotypes, classified as six categories.

| 1. **Midlife** | **The HRS** | | | | **The STR** | | | |
| --- | --- | --- | --- | --- | --- | --- | --- | --- |
|  | **N** | **Events** | **HR** | **95% CI** | **N** | **Events** | **HR** | **95% CI** |
| **Total analytical sample** | 4,233 | 362 |  | | 5,856 | 327 |  | |
| MHNW | 579 | 36 | Ref | Ref | 2,050 | 112 | Ref | Ref |
| MHOW | 901 | 50 | 0.82 | 0.53 – 1.26 | 1,354 | 80 | 1.09 | 0.82 – 1.45 |
| MHO | 839 | 57 | 0.85 | 0.56 – 1.30 | 298 | 11 | 0.73 | 0.39 – 1.36 |
| MUNW | 177 | 24 | 1.08 | 0.64 –1.83 | 582 | 40 | 1.29 | 0.89 – 1.86 |
| MUOW | 549 | 66 | 1.21 | 0.81 –1.83 | 1,040 | 54 | 0.94 | 0.67 – 1.31 |
| MUO | 1188 | 129 | 0.96 | 0.66 – 1.40 | 532 | 30 | 1.23 | 0.82 – 1.85 |
| **Male sample** | 1,715 | 161 |  | | 2,590 | 146 |  | |
| MHNW | 175 | 18 | Ref | Ref | 689 | 45 | Ref | Ref |
| MHOW | 427 | 29 | 1.00 | 0.55 –1.82 | 625 | 28 | 0.71 | 0.44 – 1.15 |
| MHO | 353 | 24 | 0.86 | 0.46 – 1.60 | 105 | 2 | 0.28 | 0.07 – 1.15 |
| MUNW | 76 | 11 | 1.03 | 0.48 – 2.20 | 246 | 17 | 1.24 | 0.71 – 2.18 |
| MUOW | 270 | 31 | 1.16 | 0.64 – 2.09 | 659 | 37 | 0.84 | 0.54 – 1.31 |
| MUO | 414 | 48 | 0.93 | 0.54 – 1.63 | 266 | 17 | 1.32 | 0.74 – 2.33 |
| **Female sample** | 2,518 | 201 |  | | 3,266 | 181 |  | |
| MHNW | 404 | 18 | Ref | Ref | 1,361 | 67 | Ref | Ref |
| MHOW | 474 | 21 | 0.68 | 0.36 –1.28 | 729 | 52 | 1.43 | 0.99 – 2.05 |
| MHO | 486 | 33 | 0.87 | 0.48 – 1.56 | 193 | 9 | 1.12 | 0.55 – 2.24 |
| MUNW | 101 | 13 | 1.21 | 0.57 – 2.54 | 336 | 23 | 1.34 | 0.83 – 2.17 |
| MUOW | 279 | 35 | 1.32 | 0.74 – 2.35 | 381 | 17 | 0.90 | 0.53 – 1.54 |
| MUO | 774 | 81 | 1.01 | 0.60 – 1.72 | 266 | 13 | 1.08 | 0.59 – 1.95 |

| 1. **Late-life** | **The HRS** | | | | **The STR** | | | |
| --- | --- | --- | --- | --- | --- | --- | --- | --- |
|  | **N** | **Events** | **HR** | **95% CI** | **N** | **Events** | **HR** | **95% CI** |
| **Total analytical sample** | 8,837 | 2,077 |  | | 7,212 | 1,669 |  | |
| MHNW | 1,152 | 284 | Ref | Ref | 2,014 | 475 | Ref | Ref |
| MHOW | 1,548 | 302 | **0.84** | **0.72 – 0.99** | 1,469 | 331 | 0.92 | 0.80 – 1.06 |
| MHO | 1,134 | 213 | 0.86 | 0.72 – 1.03 | 318 | 59 | 0.77 | 0.59 – 1.01 |
| MUNW | 727 | 238 | 1.07 | 0.89 – 1.27 | 1,034 | 264 | 1.12 | 0.97 – 1.31 |
| MUOW | 1,734 | 461 | 0.93 | 0.80 – 1.08 | 1,711 | 416 | 1.07 | 0.94 – 1.23 |
| MUO | 2,542 | 579 | 1.02 | 0.88 – 1.18 | 666 | 124 | 0.85 | 0.70 – 1.04 |
| **Male sample** | 3,759 | 847 |  | | 3,349 | 710 |  | |
| MHNW | 392 | 101 | Ref | Ref | 837 | 183 | Ref | Ref |
| MHOW | 746 | 149 | 0.85 | 0.65 – 1.09 | 674 | 131 | 0.91 | 0.73 – 1.14 |
| MHO | 505 | 88 | 0.79 | 0.59 – 1.05 | 106 | 17 | 0.81 | 0.49 – 1.33 |
| MUNW | 230 | 70 | 1.07 | 0.78 – 1.47 | 500 | 121 | 1.22 | 0.97 – 1.54 |
| MUOW | 828 | 201 | 0.87 | 0.68 – 1.11 | 959 | 212 | 1.14 | 0.94 – 1.40 |
| MUO | 1,058 | 238 | 1.06 | 0.83 –1.35 | 273 | 46 | 1.02 | 0.73 – 1.41 |

| **Female sample** | 5,078 | 1,230 |  | | 3,863 | 959 |  | |
| --- | --- | --- | --- | --- | --- | --- | --- | --- |
| MHNW | 760 | 183 | Ref | Ref | 1,177 | 292 | Ref | Ref |
| MHOW | 802 | 153 | 0.83 | 0.66 – 1.03 | 795 | 200 | 0.94 | 0.78 – 1.13 |
| MHO | 629 | 125 | 0.94 | 0.74 – 1.18 | 212 | 42 | 0.76 | 0.55 – 1.05 |
| MUNW | 497 | 168 | 1.07 | 0.86 – 1.32 | 534 | 143 | 1.07 | 0.87 – 1.30 |
| MUOW | 906 | 260 | 1.00 | 0.83 – 1.22 | 752 | 204 | 1.03 | 0.86 – 1.24 |
| MUO | 1,484 | 341 | 0.99 | 0.82 – 1.19 | 393 | 78 | 0.79 | 0.61 – 1.01 |

Hazard rate ratios (HR) and 95% confidence intervals (CI) from Cox proportional hazard regression, showing risk of dementia in relation to MHOW, MHO, MUNW, MUOW, and MUO, compared to the reference category MHNW. Results are presented for measures taken in a) midlife (≤65 years) and b) late-life (>65 years), in the total analytical sample, the male analytical sample, and the female analytical sample. All models were adjusted for age, sex, smoking status, and education level. Models of the HRS were further adjusted for ethnicity, and models of the STR for sub-study. Bold numbers indicate statistical significance at the α=0.05 level.

Abbreviations: CI – Confidence interval, HR – Hazard ratio, HRS – Health and Retirement Study, N – Number of individuals, MHNW – Metabolically healthy normal weight, MHO – Metabolically healthy obesity, MHOW – Metabolically healthy overweight, MUNW – Metabolically unhealthy normal weight, MUO – Metabolically unhealthy obesity, MUOW – Metabolically unhealthy overweight, STR – Swedish Twin Registry**.**

# Table S11. Risk of dementia in relation to obesity and metabolic health phenotypes, additionally adjusting for physical activity and alcohol consumption.

| 1. **Midlife** | **The HRS** | | | | **The STR** | | | |
| --- | --- | --- | --- | --- | --- | --- | --- | --- |
|  | **N** | **Events** | **HR** | **95% CI** | **N** | **Events** | **HR** | **95% CI** |
| **Total analytical sample** | 4,218 | 362 |  | | 5,824 | 323 |  | |
| MHNO | 1,473 | 86 | Ref | Ref | 3,387 | 189 | Ref | Ref |
| MHO | 836 | 57 | 0.94 | 0.67 – 1.32 | 297 | 11 | 0.73 | 0.39 – 1.34 |
| MUNO | 723 | 90 | 1.28 | 0.94 – 1.73 | 1,613 | 93 | 1.05 | 0.81 – 1.35 |
| MUO | 1,186 | 129 | 1.02 | 0.77 – 1.36 | 527 | 30 | 1.26 | 0.85 – 1.87 |
| **Male sample** | 1,707 | 161 |  |  | 2,579 | 144 |  |  |
| MHNO | 599 | 47 | Ref | Ref | 1,308 | 72 | Ref | Ref |
| MHO | 351 | 24 | 0.82 | 0.50 – 1.35 | 105 | 2 | 0.33 | 0.08 – 1.35 |
| MUNO | 345 | 42 | 1.04 | 0.68 – 1.60 | 902 | 53 | 1.08 | 0.76 – 1.56 |
| MUO | 412 | 48 | 0.87 | 0.57 – 1.33 | 264 | 17 | 1.62 | 0.94 – 2.79 |
| **Female sample** | 2,511 | 201 |  |  | 3,245 | 179 |  |  |
| MHNO | 874 | 39 | Ref | Ref | 2,079 | 117 | Ref | Ref |
| MHO | 485 | 33 | 1.08 | 0.67 – 1.73 | 192 | 9 | 1.02 | 0.52 – 2.02 |
| MUNO | 378 | 48 | **1.61** | **1.04 – 2.49** | 711 | 40 | 1.02 | 0.71 – 1.47 |
| MUO | 774 | 81 | 1.24 | 0.83 – 1.86 | 263 | 13 | 1.02 | 0.57 – 1.83 |

| 1. **Late-life** | **The HRS** | | | | **The STR** | | | |
| --- | --- | --- | --- | --- | --- | --- | --- | --- |
|  | **N** | **Events** | **HR** | **95% CI** | **N** | **Events** | **HR** | **95% CI** |
| **Total analytical sample** | 8,797 | 2,067 |  | | 6,717 | 1,547 |  | |
| MHNO | 2,686 | 582 | Ref | Ref | 3,151 | 725 | Ref | Ref |
| MHO | 1,128 | 211 | 0.92 | 0.78 – 1.08 | 285 | 54 | 0.81 | 0.61 – 1.07 |
| MUNO | 2,451 | 697 | 1.04 | 0.93 – 1.16 | 2,630 | 646 | **1.12** | **1.01 – 1.25** |
| MUO | 2,532 | 577 | 1.06 | 0.94 – 1.19 | 651 | 122 | 0.88 | 0.72 – 1.07 |
| **Male sample** | 3,738 | 842 |  |  | 3,165 | 667 |  |  |
| MHNO | 1,130 | 249 | Ref | Ref | 1,397 | 289 | Ref | Ref |
| MHO | 501 | 87 | 0.86 | 0.67 – 1.10 | 96 | 15 | 0.80 | 0.48 – 1.35 |
| MUNO | 1,055 | 270 | 0.96 | 0.80 – 1.15 | 1,403 | 318 | **1.22** | **1.04 – 1.43** |
| MUO | 1,052 | 236 | 1.08 | 0.89 – 1.30 | 269 | 45 | 1.05 | 0.76 – 1.44 |
| **Female sample** | 5,059 | 1,225 |  |  | 3,552 | 880 |  |  |
| MHNO | 1,556 | 333 | Ref | Ref | 1,754 | 436 | Ref | Ref |
| MHO | 627 | 124 | 0.99 | 0.81 – 1.23 | 189 | 39 | 0.79 | 0.57 – 1.10 |
| MUNO | 1,396 | 427 | 1.11 | 0.95 – 1.29 | 1,227 | 328 | 1.05 | 0.91 – 1.22 |
| MUO | 1,480 | 341 | 1.04 | 0.89 – 1.23 | 382 | 77 | 0.79 | 0.61 – 1.01 |

Hazard rate ratios (HR) and 95% confidence intervals (CI) from Cox proportional hazard regression, showing risk of dementia in relation to MUO, MUNO, and MHO compared to the reference category MHNO. Results are presented for measures taken in a) midlife (≤65 years) and b) late-life (>65 years), in the total analytical sample, the male analytical sample, and the female analytical sample. All models were adjusted for age, sex, smoking status, education level, physical activity, and alcohol consumption. Models of the HRS were further adjusted for ethnicity, and models of the STR for sub-study. Bold numbers indicate statistical significance at the α=0.05 level.

Abbreviations: CI – Confidence interval, HR – Hazard ratio, HRS – Health and Retirement Study, N – Number of individuals, MHNO – Metabolically healthy no obesity, MHO – Metabolically healthy obesity, MUNO – Metabolically unhealthy no obesity, MUO – Metabolically unhealthy obesity, STR – Swedish Twin Registry**.**

# Table S12. Risk of dementia in relation to obesity and metabolic health phenotypes, additionally adjusting for depressive symptoms, physical activity, and alcohol consumption.

| 1. **Midlife** | **The HRS** | | | | **The STR** | | | |
| --- | --- | --- | --- | --- | --- | --- | --- | --- |
|  | **N** | **Events** | **HR** | **95% CI** | **N** | **Events** | **HR** | **95% CI** |
| **Total analytical sample** | 4,218 | 362 |  | | 5,811 | 323 |  | |
| MHNO | 1,473 | 86 | Ref | Ref | 3,382 | 189 | Ref | Ref |
| MHO | 836 | 57 | 0.97 | 0.69 – 1.36 | 296 | 11 | 0.72 | 0.39 – 1.33 |
| MUNO | 723 | 90 | 1.31 | 0.97 – 1.77 | 1,607 | 93 | 1.04 | 0.80 – 1.34 |
| MUO | 1,186 | 129 | 1.05 | 0.79 – 1.39 | 526 | 30 | 1.26 | 0.85 – 1.86 |
| **Male sample** | 1,707 | 161 |  |  | 2,576 | 144 |  |  |
| MHNO | 599 | 47 | Ref | Ref | 1,306 | 72 | Ref | Ref |
| MHO | 351 | 24 | 0.88 | 0.53 – 1.44 | 105 | 2 | 0.33 | 0.08 – 1.33 |
| MUNO | 345 | 42 | 1.07 | 0.70 – 1.64 | 902 | 53 | 1.07 | 0.75 – 1.54 |
| MUO | 412 | 48 | 0.90 | 0.59 – 1.36 | 263 | 17 | 1.63 | 0.95 – 2.81 |
| **Female sample** | 2,511 | 201 |  |  | 3,235 | 179 |  |  |
| MHNO | 874 | 39 | Ref | Ref | 2,076 | 117 | Ref | Ref |
| MHO | 485 | 33 | 1.10 | 0.69 – 1.77 | 191 | 9 | 1.03 | 0.52 – 2.04 |
| MUNO | 378 | 48 | **1.66** | **1.07 – 2.56** | 705 | 40 | 1.01 | 0.70 – 1.46 |
| MUO | 774 | 81 | 1.26 | 0.84 – 1.88 | 263 | 13 | 1.00 | 0.56 – 1.80 |

| 1. **Late-life** | **The HRS** | | | | **The STR** | | | |
| --- | --- | --- | --- | --- | --- | --- | --- | --- |
|  | **N** | **Events** | **HR** | **95% CI** | **N** | **Events** | **HR** | **95% CI** |
| **Total analytical sample** | 8,797 | 2,067 |  | | 6,678 | 1,537 |  | |
| MHNO | 2,686 | 582 | Ref | Ref | 3,138 | 722 | Ref | Ref |
| MHO | 1,128 | 211 | 0.88 | 0.75 – 1.04 | 283 | 53 | 0.79 | 0.60 – 1.05 |
| MUNO | 2,451 | 697 | 1.00 | 0.90 – 1.12 | 2,608 | 640 | 1.12 | 1.01 – 1.25 |
| MUO | 2,532 | 577 | 1.02 | 0.90 – 1.15 | 649 | 122 | 0.87 | 0.72 – 1.06 |
| **Male sample** | 3,738 | 842 |  |  | 3,152 | 665 |  |  |
| MHNO | 1,130 | 249 | Ref | Ref | 1,395 | 288 | Ref | Ref |
| MHO | 501 | 87 | 0.85 | 0.66 – 1.09 | 96 | 15 | 0.81 | 0.48 –1.36 |
| MUNO | 1,055 | 270 | 0.94 | 0.79 – 1.13 | 1,392 | 317 | **1.22** | **1.04 – 1.44** |
| MUO | 1,052 | 236 | 1.06 | 0.88 – 1.28 | 269 | 45 | 1.05 | 0.76 – 1.44 |
| **Female sample** | 5,059 | 1,225 |  |  | 3,526 | 872 |  |  |
| MHNO | 1,556 | 333 | Ref | Ref | 1,743 | 434 | Ref | Ref |
| MHO | 627 | 124 | 0.95 | 0.77 – 1.17 | 187 | 38 | 0.76 | 0.55 – 1.07 |
| MUNO | 1,396 | 427 | 1.06 | 0.91 – 1.23 | 1,216 | 323 | 1.04 | 0.90 – 1.21 |
| MUO | 1,480 | 341 | 1.00 | 0.85 – 1.18 | 380 | 77 | 0.78 | 0.61 – 1.00 |

Hazard rate ratios (HR) and 95% confidence intervals (CI) from Cox proportional hazard regression, showing risk of dementia in relation to MUO, MUNO, and MHO compared to the reference category MHNO. Results are presented for measures taken in a) midlife (≤65 years) and b) late-life (>65 years), in the total analytical sample, the male analytical sample, and the female analytical sample. All models were adjusted for age, sex, smoking status, education level, physical activity, alcohol consumption, and , depressive symptoms. Models of the HRS were further adjusted for ethnicity, and models of the STR for sub-study. Bold numbers indicate statistical significance at the α=0.05 level.

Abbreviations: CI – Confidence interval, HR – Hazard ratio, HRS – Health and Retirement Study, N – Number of individuals, MHNO – Metabolically healthy no obesity, MHO – Metabolically healthy obesity, MUNO – Metabolically unhealthy no obesity, MUO – Metabolically unhealthy obesity, STR – Swedish Twin Registry**.**

# Table S13. Risk of dementia in relation to obesity and metabolic health phenotypes, additionally adjusting for depressive symptoms, physical activity, alcohol consumption, and Apolipoprotein E ε4.

| 1. **Midlife** | **The HRS** | | | | **The STR** | | | |
| --- | --- | --- | --- | --- | --- | --- | --- | --- |
|  | **N** | **Events** | **HR** | **95% CI** | **N** | **Events** | **HR** | **95% CI** |
| **Total analytical sample** | 3,877 | 335 |  | | 4,992 | 283 |  | |
| MHNO | 1,375 | 80 | Ref | Ref | 2,851 | 159 | Ref | Ref |
| MHO | 776 | 53 | 0.97 | 0.68 –1.38 | 257 | 9 | 0.71 | 0.36 – 1.40 |
| MUNO | 645 | 81 | 1.27 | 0.93 –1.75 | 1,409 | 87 | 1.03 | 0.78 – 1.35 |
| MUO | 1,081 | 121 | 1.05 | 0.78 – 1.40 | 475 | 28 | 1.27 | 0.84 – 1.91 |
| **Male sample** | 1,562 | 146 |  |  | 2,241 | 136 |  |  |
| MHNO | 561 | 45 | Ref | Ref | 1,109 | 65 | Ref | Ref |
| MHO | 319 | 21 | 0.82 | 0.48 – 1.39 | 92 | 2 | 0.35 | 0.08 – 1.43 |
| MUNO | 301 | 36 | 0.98 | 0.62 – 1.53 | 802 | 53 | 1.03 | 0.71 – 1.50 |
| MUO | 381 | 44 | 0.87 | 0.57 – 1.34 | 238 | 16 | 1.58 | 0.90 – 2.77 |
| **Female sample** | 2,315 | 189 |  |  | 2,751 | 147 |  |  |
| MHNO | 814 | 35 | Ref | Ref | 1,742 | 94 | Ref | Ref |
| MHO | 457 | 32 | 1.17 | 0.72 – 1.92 | 165 | 7 | 1.09 | 0.50 – 2.36 |
| MUNO | 344 | 45 | **1.72** | **1.09 – 2.72** | 607 | 34 | 1.07 | 0.72 – 1.59 |
| MUO | 700 | 77 | 1.34 | 0.88 – 2.04 | 237 | 12 | 0.99 | 0.53 – 1.82 |

| 1. **Late-life** | **The HRS** | | | | **The STR** | | | |
| --- | --- | --- | --- | --- | --- | --- | --- | --- |
|  | **N** | **Events** | **HR** | **95% CI** | **N** | **Events** | **HR** | **95% CI** |
| **Total analytical sample** | 8,220 | 1,938 |  | | 5,649 | 1,307 |  | |
| MHNO | 2,528 | 554 | Ref | Ref | 2,636 | 614 | Ref | Ref |
| MHO | 1,067 | 205 | 0.92 | 0.78 – 1.09 | 233 | 46 | 0.80 | 0.59 – 1.08 |
| MUNO | 2,266 | 644 | 0.96 | 0.86 – 1.08 | 2,211 | 540 | 1.08 | 0.96 – 1.21 |
| MUO | 2,359 | 535 | 0.99 | 0.87 – 1.12 | 569 | 107 | 0.85 | 0.69 – 1.05 |
| **Male sample** | 3,500 | 785 |  |  | 2,853 | 599 |  |  |
| MHNO | 1,064 | 238 | Ref | Ref | 1,255 | 254 | Ref | Ref |
| MHO | 484 | 84 | 0.84 | 0.66 – 1.09 | 89 | 15 | 0.89 | 0.53 – 1.50 |
| MUNO | 973 | 242 | 0.87 | 0.72 – 1.05 | 1,262 | 287 | **1.21** | **1.02 – 1.44** |
| MUO | 979 | 221 | 1.01 | 0.83 – 1.22 | 247 | 43 | 1.10 | 0.80 – 1.53 |
| **Female sample** | 4,720 | 1,153 |  |  | 2,796 | 708 |  |  |
| MHNO | 1,464 | 316 | Ref | Ref | 1,381 | 360 | Ref | Ref |
| MHO | 583 | 121 | 1.04 | 0.84 – 1.29 | 144 | 31 | 0.74 | 0.51 – 1.07 |
| MUNO | 1,293 | 402 | 1.04 | 0.89 – 1.21 | 949 | 253 | 0.97 | 0.82 – 1.14 |
| MUO | 1,380 | 314 | 0.98 | 0.83 – 1.17 | 322 | 64 | **0.73** | **0.56 – 0.96** |

Hazard rate ratios (HR) and 95% confidence intervals (CI) from Cox proportional hazard regression, showing risk of dementia in relation to MUO, MUNO, and MHO compared to the reference category MHNO. Results are presented for measures taken in a) midlife (≤65 years) and b) late-life (>65 years), in the total analytical sample, the male analytical sample, and the female analytical sample. All models were adjusted for age, sex, smoking status, education level, depression, physical activity, alcohol consumption, and Apolipoprotein E ε4. Models of the HRS were further adjusted for ethnicity, and models of the STR for sub-study. Bold numbers indicate statistical significance at the α=0.05 level.

Abbreviations: CI – Confidence interval, HR – Hazard ratio, HRS – Health and Retirement Study, N – Number of individuals, MHNO – Metabolically healthy no obesity, MHO – Metabolically healthy obesity, MUNO – Metabolically unhealthy no obesity, MUO – Metabolically unhealthy obesity, STR – Swedish Twin Registry**.**

# Table S14. Risk of dementia in relation to obesity and metabolic health phenotypes, from competing risk regression modelling death as the competing event

| 1. **Midlife** | **The HRS** | | | | | **The STR** | | | | |
| --- | --- | --- | --- | --- | --- | --- | --- | --- | --- | --- |
|  | **N** | **Events** | **Death** | **HR** | **95% CI** | **N** | **Death** | **Events** | **HR** | **95% CI** |
| **Total analytical sample** | 4,233 | 362 | 265 |  | | 5,855 | 327 | 534 |  | |
| MHNO | 1,480 | 86 | 62 | Ref | Ref | 3,404 | 192 | 250 | Ref | Ref |
| MHO | 839 | 57 | 39 | 0.96 | 0.68 – 1.35 | 298 | 11 | 28 | 0.68 | 0.37 – 1.26 |
| MUNO | 726 | 90 | 62 | 1.24 | 0.92 – 1.67 | 1,621 | 94 | 178 | 0.99 | 0.77 – 1.28 |
| MUO | 1,188 | 129 | 102 | 1.05 | 0.79 – 1.38 | 532 | 30 | 78 | 1.08 | 0.74 – 1.59 |

| 1. **Late-life** | **The HRS** | | | | | **The STR** | | | | |
| --- | --- | --- | --- | --- | --- | --- | --- | --- | --- | --- |
|  | **N** | **Events** | **Death** | **HR** | **95% CI** | **N** | **Death** | **Events** | **HR** | **95% CI** |
| **Total analytical sample** | 8,895 | 2,078 | 2,075 |  | | 7,143 | 1,669 | 2,660 |  | |
| MHNO | 2,721 | 586 | 590 | Ref | Ref | 3,451 | 806 | 1,193 | Ref | Ref |
| MHO | 1,140 | 213 | 227 | **0.78** | **0.68 – 0.90** | 314 | 59 | 134 | **0.70** | **0.53 – 0.92** |
| MUNO | 2,477 | 700 | 640 | 1.01 | 0.92 – 1.12 | 2,719 | 680 | 1,050 | 1.06 | 0.95 – 1.18 |
| MUO | 2,557 | 579 | 618 | **0.78** | **0.71 – 0.87** | 659 | 124 | 283 | **0.72** | **0.59 – 0.88** |

Hazard rate ratios (HR) and 95% confidence intervals (CI) from Fine-Gray subdistribution hazards regression, showing risk of dementia in relation to MUO, MUNO, and MHO compared to the reference category MHNO. Results are presented for measures taken in a) midlife (≤65 years) and b) late-life (>65 years) in the total analytical sample. All models were adjusted for age, sex, smoking status, education level, depression, physical activity, alcohol consumption, and Apolipoprotein E ε4. Models of the HRS were further adjusted for ethnicity, and models of the STR for sub-study. Bold numbers indicate statistical significance at the α=0.05 level.

Abbreviations: CI – Confidence interval, HR – Hazard ratio, HRS – Health and Retirement Study, N – Number of individuals, MHNO – Metabolically healthy no obesity, MHO – Metabolically healthy obesity, MUNO – Metabolically unhealthy no obesity, MUO – Metabolically unhealthy obesity, STR – Swedish Twin Registry**.**
